# Supplementary material for: Reactive-site-centric chemoproteomics identifies a distinct class of deubiquitinase enzymes
Source: Nat Commun. 2018 Mar 21;9:1162. doi: 10.1038/s41467-018-03511-6 (PMC5862848; doi:10.1038/s41467-018-03511-6)
Supplement: Supplementary file 1 — Supplementary Information(DOCX 17840 kb) [file 41467_2018_3511_MOESM1_ESM.docx]

**Reactive-site-centric chemoproteomics identifies a distinct class of deubiquitinase enzymes**

*Supplementary Information*

David S. Hewings, Johanna Heideker, Taylur P. Ma, Andrew Ah Young, Farid El Oualid, Alessia Amore, Gregory T. Costakes, Daniel Kirchhofer, Bradley Brasher, Thomas Pillow, Nataliya Popovych, Till Maurer, Carsten Schwerdtfeger, William F. Forrest, Kebing Yu, John Flygare, Matthew Bogyo, Ingrid E. Wertz

Pages 2-5: Supplementary Tables 1-5

Pages 6-29: Supplementary Figures 1-26

Pages 30-43: Supplementary Methods

Page 44: Supplementary References

| **Gene name** | **Uniprot ID** | **Class** | **Length (amino acids)** | **DUB activity reported?** |
| --- | --- | --- | --- | --- |
| ATXN3 | ATX3_HUMAN | Josephin | 364 | Yes |
| ATXN3L | ATX3L_HUMAN | Josephin | 355 | Yes |
| JOSD1 | JOS1_HUMAN | Josephin | 202 | Yes |
| JOSD2 | JOS2_HUMAN | Josephin | 188 | Yes |
| BRCC3 | BRCC3_HUMAN | MPN+ | 316 | Yes |
| COPS5 | CSN5_HUMAN | MPN+ | 334 | Yes |
| COPS6 | CSN6_HUMAN | MPN+ | 327 | No |
| EIF3F | EIF3F_HUMAN | MPN+ | 357 | Yes |
| MPND | MPND_HUMAN | MPN+ | 471 | Yes |
| MYSM1 | MYSM1_HUMAN | MPN+ | 828 | Yes |
| PRPF8 | PRP8_HUMAN | MPN+ | 2335 | No |
| PSMD14 | PSDE_HUMAN | MPN+ | 310 | Yes |
| STAMBP | STABP_HUMAN | MPN+ | 424 | Yes |
| STAMBPL1 | STALP_HUMAN | MPN+ | 436 | Yes |
| TNFAIP3 | TNAP3_HUMAN | OTU | 790 | Yes |
| ALG13 | ALG13_HUMAN | OTU | 1137 | Yes |
| YOD1 | OTU1_HUMAN | OTU | 348 | Yes |
| OTUB1 | OTUB1_HUMAN | OTU | 271 | Yes |
| OTUB2 | OTUB2_HUMAN | OTU | 234 | Yes |
| OTUD1 | OTUD1_HUMAN | OTU | 481 | Yes |
| OTUD3 | OTUD3_HUMAN | OTU | 398 | Yes |
| OTUD4 | OTUD4_HUMAN | OTU | 1114 | Yes |
| OTUD5 | OTUD5_HUMAN | OTU | 571 | Yes |
| OTUD6A | OTU6A_HUMAN | OTU | 288 | Yes |
| OTUD6B | OTU6B_HUMAN | OTU | 293 | Yes |
| OTUD7A | OTU7A_HUMAN | OTU | 926 | Yes |
| OTUD7B | OTU7B_HUMAN | OTU | 843 | Yes |
| OTULIN | OTUL_HUMAN | OTU | 352 | Yes |
| VCPIP1 | VCIP1_HUMAN | OTU | 1222 | Yes |
| ZRANB1 | ZRAN1_HUMAN | OTU | 708 | Yes |
| BAP1 | BAP1_HUMAN | UCH | 729 | Yes |
| UCHL1 | UCHL1_HUMAN | UCH | 223 | Yes |
| UCHL3 | UCHL3_HUMAN | UCH | 230 | Yes |
| UCHL5 | UCHL5_HUMAN | UCH | 329 | Yes |
| CYLD | CYLD_HUMAN | USP | 956 | Yes |
| PAN2 | PAN2_HUMAN | USP | 1202 | No |
| USP1 | UBP1_HUMAN | USP | 785 | Yes |
| USP10 | UBP10_HUMAN | USP | 798 | Yes |
| USP11 | UBP11_HUMAN | USP | 963 | Yes |
| USP12 | UBP12_HUMAN | USP | 370 | Yes |
| USP13 | UBP13_HUMAN | USP | 863 | Yes |
| USP14 | UBP14_HUMAN | USP | 494 | Yes |
| USP15 | UBP15_HUMAN | USP | 981 | Yes |
| USP16 | UBP16_HUMAN | USP | 823 | Yes |
| USP17L24 | U17LO_HUMAN | USP | 530 | Yes |
| USP17L1 | U17L1_HUMAN | USP | 530 | Yes |
| USP17L2 | U17L2_HUMAN | USP | 530 | Yes |

| **Gene name** | **Uniprot ID** | **Class** | **Length (amino acids)** | **DUB activity reported?** |
| --- | --- | --- | --- | --- |
| USP18 | UBP18_HUMAN | USP | 372 | No |
| USP19 | UBP19_HUMAN | USP | 1318 | Yes |
| USP2 | UBP2_HUMAN | USP | 605 | Yes |
| USP20 | UBP20_HUMAN | USP | 914 | Yes |
| USP21 | UBP21_HUMAN | USP | 565 | Yes |
| USP22 | UBP22_HUMAN | USP | 525 | Yes |
| USP24 | UBP24_HUMAN | USP | 2620 | Yes |
| USP25 | UBP25_HUMAN | USP | 1055 | Yes |
| USP26 | UBP26_HUMAN | USP | 913 | Yes |
| USP27X | UBP27_HUMAN | USP | 438 | Yes |
| USP28 | UBP28_HUMAN | USP | 1077 | Yes |
| USP29 | UBP29_HUMAN | USP | 922 | Yes |
| USP3 | UBP3_HUMAN | USP | 520 | Yes |
| USP30 | UBP30_HUMAN | USP | 517 | Yes |
| USP31 | UBP31_HUMAN | USP | 1352 | Yes |
| USP32 | UBP32_HUMAN | USP | 1604 | Yes |
| USP33 | UBP33_HUMAN | USP | 942 | Yes |
| USP34 | UBP34_HUMAN | USP | 3546 | Yes |
| USP35 | UBP35_HUMAN | USP | 1018 | Yes |
| USP36 | UBP36_HUMAN | USP | 1121 | Yes |
| USP37 | UBP37_HUMAN | USP | 979 | Yes |
| USP38 | UBP38_HUMAN | USP | 1042 | Yes |
| USP39 | SNUT2_HUMAN | USP | 565 | No |
| USP4 | UBP4_HUMAN | USP | 963 | Yes |
| USP40 | UBP40_HUMAN | USP | 1235 | Yes |
| USP41 | UBP41_HUMAN | USP | 358 | Yes |
| USP42 | UBP42_HUMAN | USP | 1324 | Yes |
| USP43 | UBP43_HUMAN | USP | 1123 | Yes |
| USP44 | UBP44_HUMAN | USP | 712 | Yes |
| USP45 | UBP45_HUMAN | USP | 814 | Yes |
| USP46 | UBP46_HUMAN | USP | 366 | Yes |
| USP47 | UBP47_HUMAN | USP | 1375 | Yes |
| USP48 | UBP48_HUMAN | USP | 1035 | Yes |
| USP49 | UBP49_HUMAN | USP | 688 | Yes |
| USP5 | UBP5_HUMAN | USP | 858 | Yes |
| USP50 | UBP50_HUMAN | USP | 339 | No |
| USP51 | UBP51_HUMAN | USP | 711 | Yes |
| USP53 | UBP53_HUMAN | USP | 1073 | Yes |
| USP54 | UBP54_HUMAN | USP | 1684 | No |
| USP6 | UBP6_HUMAN | USP | 1406 | Yes |
| USP7 | UBP7_HUMAN | USP | 1102 | Yes |
| USP8 | UBP8_HUMAN | USP | 1118 | Yes |
| USP9X | USP9X_HUMAN | USP | 2570 | Yes |
| USP9Y | USP9Y_HUMAN | USP | 2555 | Yes |
| MINDY3 | F188A_HUMAN | MINDY | 445 | Yes |
| MINDY4 | F188B_HUMAN | MINDY | 757 | Yes |
| MINDY1 | FA63A_HUMAN | MINDY | 469 | Yes |
| MINDY2 | FA63B_HUMAN | MINDY | 621 | Yes |

**Supplementary Table 1 | List of known human deubiquitinases considered in this study**.

| Peptide | Protein | Residue | Total PSMs | | |
| --- | --- | --- | --- | --- | --- |
|  |  |  | Ub-**VS** | Ub-**VME** | Ub-**PA** |
| K.AITSGGITYQDQPWHADCFVCVTC#SK.K | FHL1 | C191 | 0 | 2 | 0 |
| R.LLPC#LHSACSACLGPAAPAAANSSGDGGAAGDGTVVDC*PVC*K.Q | TIF1B | C83 | 0 | 2 | 0 |
| R.C#GESGHLAK.D | CNBP | C57 | 2 | 0 | 0 |
| K.RC*GDSHPESPVGFGHM@STTGC#VLNK.L | CEPT1 | C11 | 0 | 0 | 1 |
| K.DVDGNDLLSYWPALGEC#EAAPC#ALQTWGSER.R | CELR3 | C2958 | 0 | 0 | 1 |
| K.GNSLTPC#ASR.V | O95662 | C189 | 0 | 0 | 1 |
| -.M@EAIGHAGTC#LGILANDGVLLAAER.R | Q7Z474 | C10 | 0 | 0 | 1 |
| R.CRVTSC#SCTCGAGAK.W | K0913 | C185 | 0 | 0 | 1 |
| R.AVC#M@LSNTTAIAEAWAR.L | TBA1A | C376 | 1 | 0 | 0 |
| K.HEPLVLFCESCDTLTC#R.D | TIF1B | C229 | 0 | 1 | 0 |
| R.C#GETGHVAINC*SK.T | CNBP | C140 | 1 | 0 | 0 |

**Supplementary Table 2 | Labeled peptides for non-DUB proteins detected following anti-HA IP.** HEK 293T cell lysate (5 mg) was treated with the indicated HA-tagged probe for 1 h. HA-labeled proteins were enriched by anti-HA immunoprecipitation, then digested by trypsin and subject to LC-MS/MS. Peptides were searched using the probe fragment as a variable modification on Cys. # = Ub-probe fragment; * = carbamidomethyl modification; @ = methionine oxidation

| Protein | Description | On-bead trypsin digestion | | Acid elution | |
| --- | --- | --- | --- | --- | --- |
|  |  | (i) | (ii) | (i) | (ii) |
| UCHL3 | Ubiquitin carboxyl-terminal hydrolase isozyme L3 | 2 (1) | 2 (1) | 0 (0) | 0 (0) |
| UCHL1 | Ubiquitin carboxyl-terminal hydrolase isozyme L1 | 1 (1) | 1 (1) | 0 (0) | 0 (0) |
| UBP48 | Ubiquitin carboxyl-terminal hydrolase 48 | 0 (0) | 1 (1) | 0 (0) | 0 (0) |

**Supplementary Table 3 | Labeled peptides arising from VPE probe hydrolysis.** Shown are spectral counts of labeled peptides arising from VPE probe ester hydrolysis, with unique peptides shown in brackets. The modification was specified as a variable modification on Cys. 5 mg HEK 293T lysate was treated with Ub-VPE probe (6.6 µg) and tagged with an acid-cleavable biotin linker by Cu-catalyzed cycloaddition. Labeled proteins were enriched on streptavidin beads then subject to on-bead trypsin digestion followed by elution with 2% formic acid.

| Protein | Description | DADPS linker | | DDE linker | |
| --- | --- | --- | --- | --- | --- |
|  |  | (i) | (ii) | (i) | (ii) |
| UCHL1 | Ubiquitin carboxyl-terminal hydrolase isozyme L1 | 1 (1) | 1 (1) | 5 (1) | 3 (2) |
| UCHL3 | Ubiquitin carboxyl-terminal hydrolase isozyme L3 | 2 (1) | 3 (1) | 3 (2) | 1 (1) |
| USP9X | Probable ubiquitin carboxyl-terminal hydrolase FAF-X | 0 (0) | 0 (0) | 2 (1) | 3 (2) |
| OTU6B | OTU domain-containing protein 6B | 0 (0) | 0 (0) | 2 (1) | 1 (1) |
| UBP5 | Ubiquitin carboxyl-terminal hydrolase 5 | 0 (0) | 0 (0) | 1 (1) | 2 (1) |
| UBP7 | Ubiquitin carboxyl-terminal hydrolase 7 | 0 (0) | 0 (0) | 2 (1) | 0 (0) |
| OTUB2 | Ubiquitin thioesterase OTUB2 | 0 (0) | 2 (1) | 0 (0) | 0 (0) |
| OTUD4 | OTU domain-containing protein 4 | 0 (0) | 1 (1) | 0 (0) | 0 (0) |

**Supplementary Table 4 | Labeled peptides arising from premature linker cleavage during on-bead digestion.** Shown are spectral counts of labeled peptides arising from linker cleavage found in the on-bead digestion fraction, with the number of unique peptides in brackets. Probe labeling was specified as a variable modification on Cys. 5 mg HEK 293T lysate was treated with Ub-VPS probe (6.6 µg) and tagged with biotin-DAPDS-azide or biotin-DDE-azide by Cu-catalyzed cycloaddition. Labeled proteins were enriched on streptavidin beads then subject to on-bead trypsin digestion. i and ii represent two biological replicates. No DDE-labeled peptides were detected after linker subsequent linker treatment, in contrast to DADPS-labeled peptides (Figure 3, Table 1).

|  | **Trypsin** | | | **Trypsin-Lys-C** | | | **Trypsin + Glu-C** | | | **Trypsin + Chymo.** | | |
| --- | --- | --- | --- | --- | --- | --- | --- | --- | --- | --- | --- | --- |
|  | i | ii | **Total** | i | ii | **Total** | i | ii | **Total** | i | ii | **Total** |
| Peptides | 104 | 80 | **184** | 132 | 161 | **293** | 176 | 130 | **306** | 163 | 138 | **301** |
| Unique sites | 39 | 32 | **47** | 50 | 54 | **69** | 50 | 35 | **55** | 41 | 32 | **42** |
| Unique sites (≥2 peptides) | 23 | 15 | **29** | 32 | 29 | **44** | 30 | 22 | **39** | 26 | 22 | **34** |

**Supplementary Table 5 | Labeled peptide counts and unique labeled sites identified by on-bead digestion followed by linker cleavage, in optimization studies.** Peptide-spectrum matches (PSMs) of Ub-VPS-probe-modified peptides arising form 1 mg protein input, after digestion on-bead with one or more proteases and DADPS linker cleavage. i and ii represent two biological replicates. For the total numbers of unique sites, or total numbers of unique sites identified with ≥2 peptides, the two replicate are combined (i.e. labeling sites identified in both replicates are counted only once.)

**Supplementary Figure 1 | Synthesis of (*E*)-3-(pent-4-yn-1-ylsulfonyl)prop-2-en-1-ammonium trifluoroacetate (VPS electrophile).** Full experimental procedures and characterization are provided in Supplementary Methods.

**Supplementary Figure 2 | Synthesis of (*E*)-4-oxo-4-(pent-4-yn-1-yloxy)but-2-en-1-ammonium chloride (VPE electrophile).** Full experimental procedures and characterization are provided in Supplementary Methods.


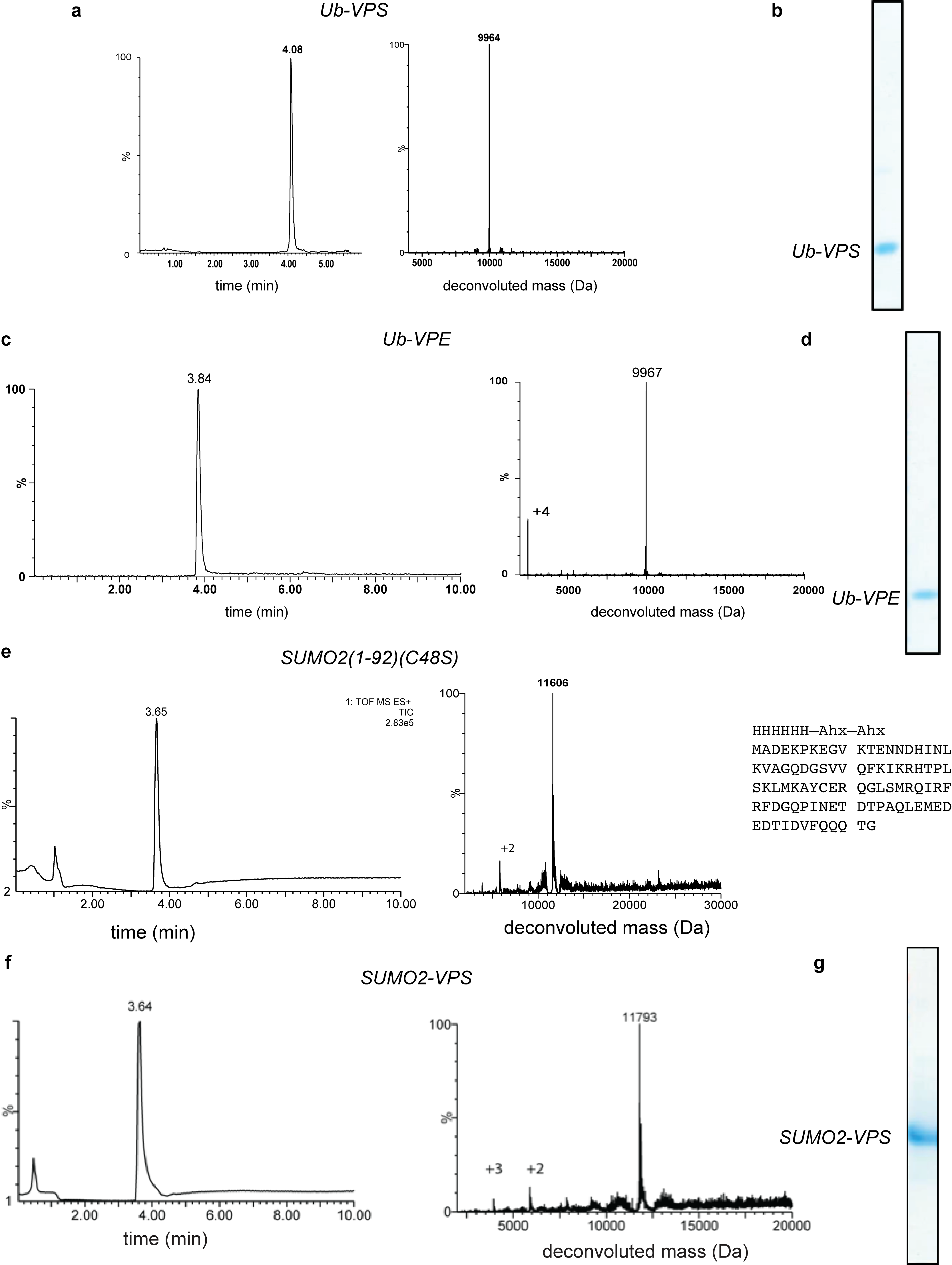


**Supplementary Figure 3 | Characterization of Ub-VPE, Ub-VPS, SUMO2(1-92)(C48S) and SUMO2-VPS.** Ub-VPS (HA-Ahx-Ahx-Ub(1-75)(Met1Nle)-VPS): (**a**) LC-MS analysis: Gradient 30-60%B over 3.5 min. (**b**) SDS-PAGE analysis. Ub-VPE (HA-Ahx-Ahx-Ub(1-75)(Met1Nle)-VPE): (**c**) LC-MS analysis: Gradient 30-60%B over 6.5 min. (**d**) SDS-PAGE analysis. Note that to eliminate Met1 oxidation, Met1 is replaced by norleucine, a well validated Met mimic^1^.

His6-Ahx-Ahx-SUMO2(1-92)(C48S): **(e)** LC-MS analysis: Gradient 50-90%B over 6.5 min. SUMO2-VPS (His6-Ahx-Ahx-SUMO2(C48S)-VPS): (**f**). LC-MS analysis: Gradient 50-90%B over 6.5 min. (**g**) SDS-PAGE analysis.

**
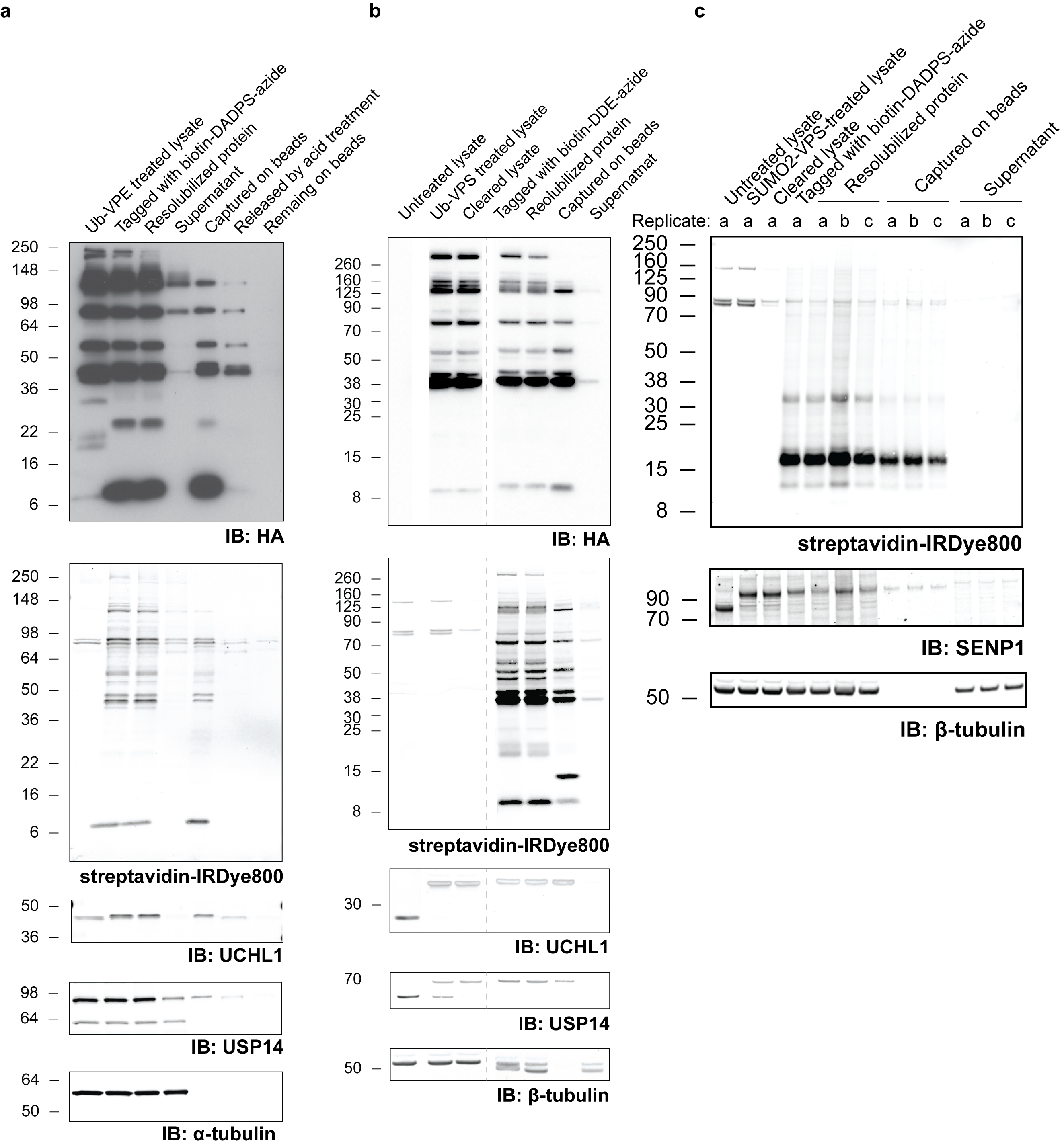
**

**Supplementary Figure 4 | Labeling, enrichment and elution of proteins with alternative probes and linkers.** (**a**) With Ub-VPE probe and DADPS linker. HEK 293T lysate (5 mg) was treated with Ub-VPE probe (6.6 µg) for 1 h. The treated lysate was cleared with streptavidin beads, and then tagged with biotin-DADPS-azide by copper-catalyzed azide-alkyne cycloaddition. Excess biotin-DADPS-azide was removed by chloroform-methanol precipitation of the proteins, and the pellet was resolubilized in 2% SDS/PBS. The tagged proteins were enriched on streptavidin beads and washed extensively. An aliquot of beads was boiled in reducing LDS sample buffer to assess enrichment, and compared to proteins that remained in the supernatant after enrichment. Proteins were eluted by treatment with 2% formic acid in water followed by washing with 1% SDS. Any proteins remaining on the beads were eluted by boiling in reducing LDS sample buffer. Proteins at each stage were analyzed by immunoblot or with a streptavidin-IRDye800 conjugate. The HA blot indicates labeled proteins; the streptavidin blot indicates labeled proteins tagged with biotin; UCHL1 and USP14 are two DUBs that are labeled and enriched in this method, and α-tubulin is a non-DUB protein which is not significantly enriched by Ub-VPE. (**b**) With Ub-VPS probe and DDE linker. As for (a), using biotin-DDE-azide linker, omitting formic acid elution. (**c**) With SUMO2-VPS probe and DADPS linker. As for (a), using SUMO2-VPS probe, omitting formic acid elution. SENP1 is a deSUMOylase that is labeled and enriched in this method.


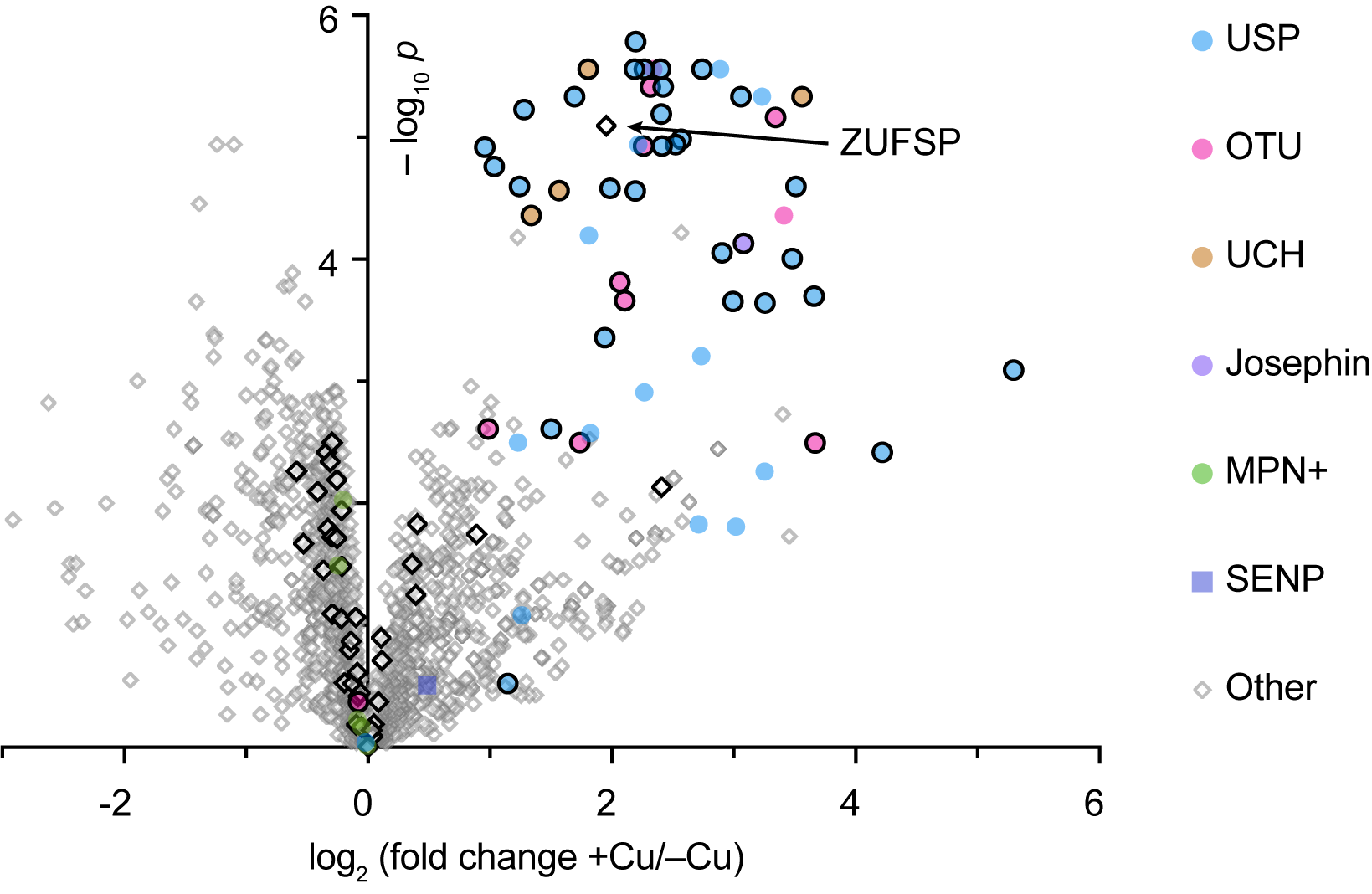


**Supplementary Figure 5 | Enrichment of proteins by Ub-VPS relative to ‘no biotinylation’ negative control.** Volcano plot of pairwise comparison of proteins in on-bead digestion fraction relative to control in which copper is omitted during reaction with biotin-DADPS-azide. Significance (−log_10_ *p*) and fold enrichment (average log_2_) are calculated from three biological replicates. A black border indicates proteins for which high-confidence probe labeling sites were identified. ZUFSP is indicated with an arrow.


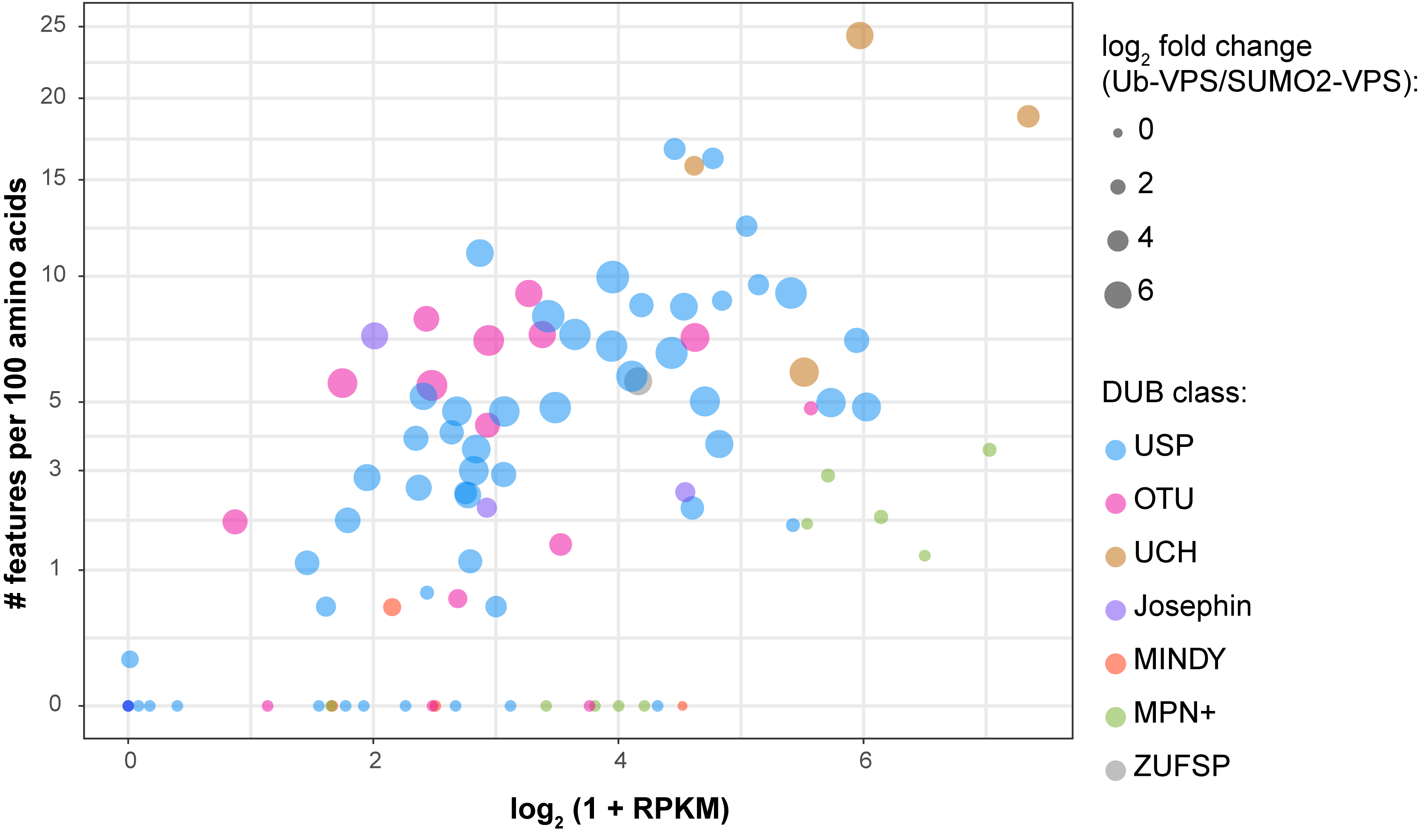


**Supplementary Figure 6 | Comparison of DUB expression and detection in HEK 293T cells.**

Shown is the number of features detected (i.e. unique peptide forms with a distinct charge state detected in the instrument) with the Ub-VPS probe normalized to protein length in units of 100 amino acids, plotted against the log_2_-normalized transcript RPKM estimates (from RNA-Seq). The radius of the point is sized in proportion to the absolute log_2_ fold change reported from *voom* between the number of features detected with Ub-VPS and SUMO2-VPS. Small points with non-zero # features therefore indicate DUBs with similar enrichment by the two probes. Full data is presented in Supplementary Data 3.


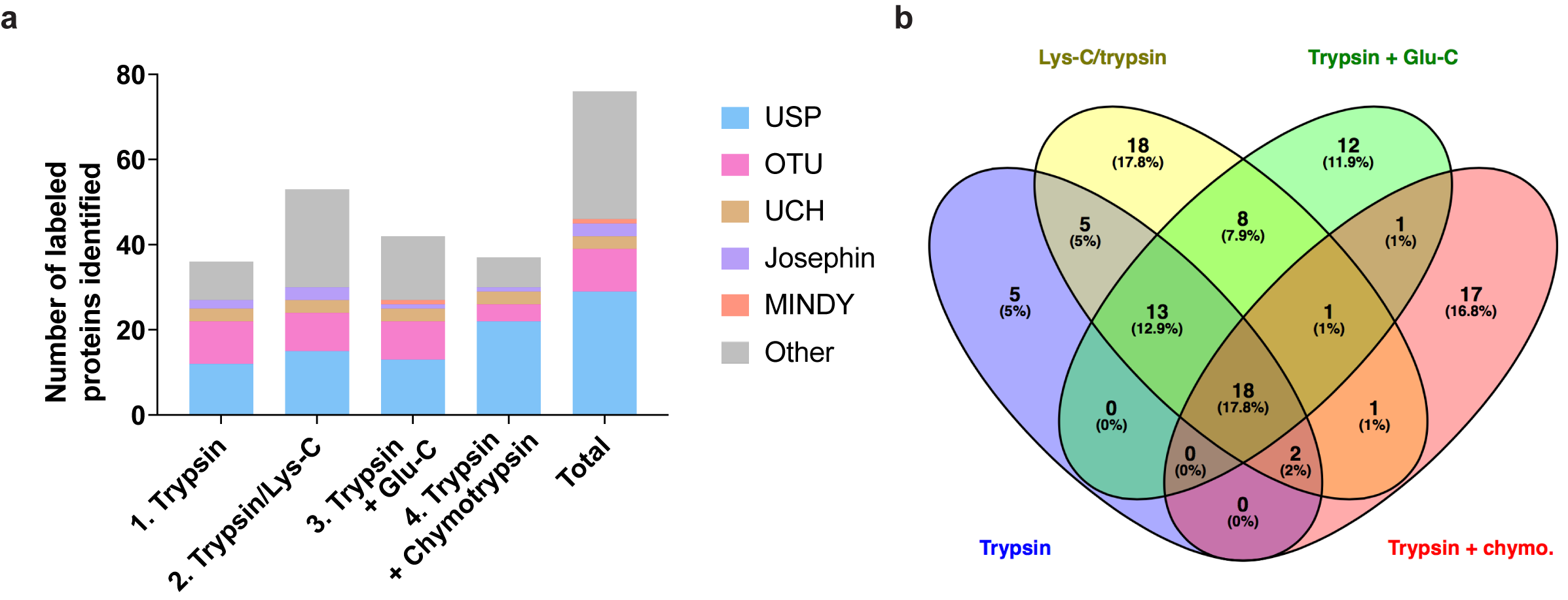


**Supplementary Figure 7 | Optimization of digestion conditions for detection of probe-modified peptides.** (**a**) Comparison of the number of unique labeled peptides identified after cleavage of DADPS linker using different combinations of proteases. Following labeling with Ub-VPS, tagging with biotin-DADPS-azide, streptavidin-biotin enrichment and on-bead digestion with trypsin or Lys-C/trypsin, labeled peptides were released by formic acid treatment (conditions 1 and 2). Alternatively, following on-bead trypsin digestion, the beads were treated with a second protease before linker cleavage (conditions 3-4). Shown is the number of proteins for which labeled peptides were detected in either one of two replicates. Each replicate corresponds to a nominal protein input of 1 mg. (**b**) Venn diagram representing the intersections of probe-labeling sites that are identified using different combinations of proteases. Diagram produced using Venny 2.1 (<http://bioinfogp.cnb.csic.es/tools/venny/>).


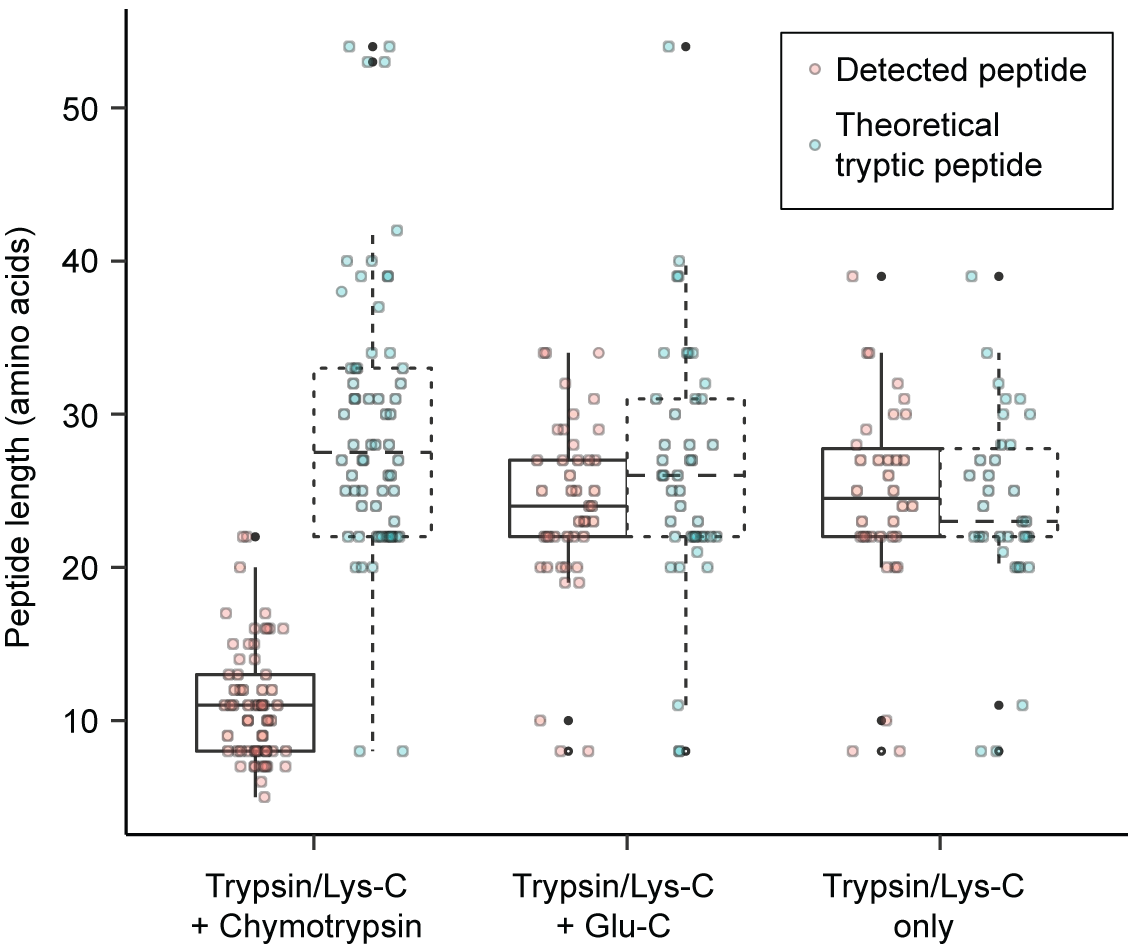


**Supplementary Figure 8 | Distribution of detected DUB active-site peptide lengths under various proteolytic conditions.** Pink dots (solid box plot) represent the DUB active-site probe-labeled peptides identified by LC-MS/MS experiments. Blue dots (dotted box plot) indicate the length of the theoretical, fully-cleaved tryptic peptides spanning the same labeled Cys residues that are detected by the indicated protease combination. Centre line, bonds of box, and whisker length indicate median, quartiles, and 150% interquartile range, respectively.

**Supplementary Figure 9 | Annotated MS/MS spectra supporting the identification of UCHL1 labeling sites.** Labeled Cys residue indicated by #.


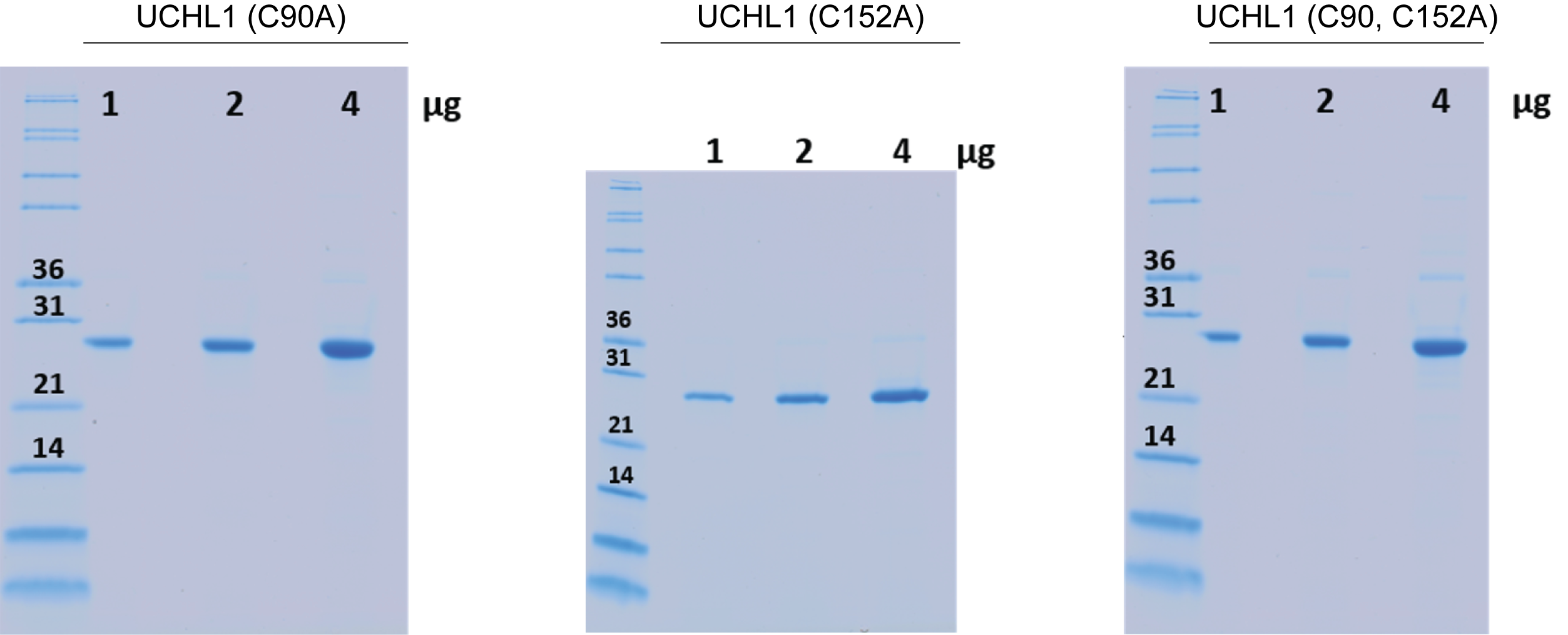


**Supplementary Figure 10 | Coomassie-stained gels of purified UCHL1 mutants**. The amount of protein shown was run on SDS-PAGE gels and detected by Coomassie staining.


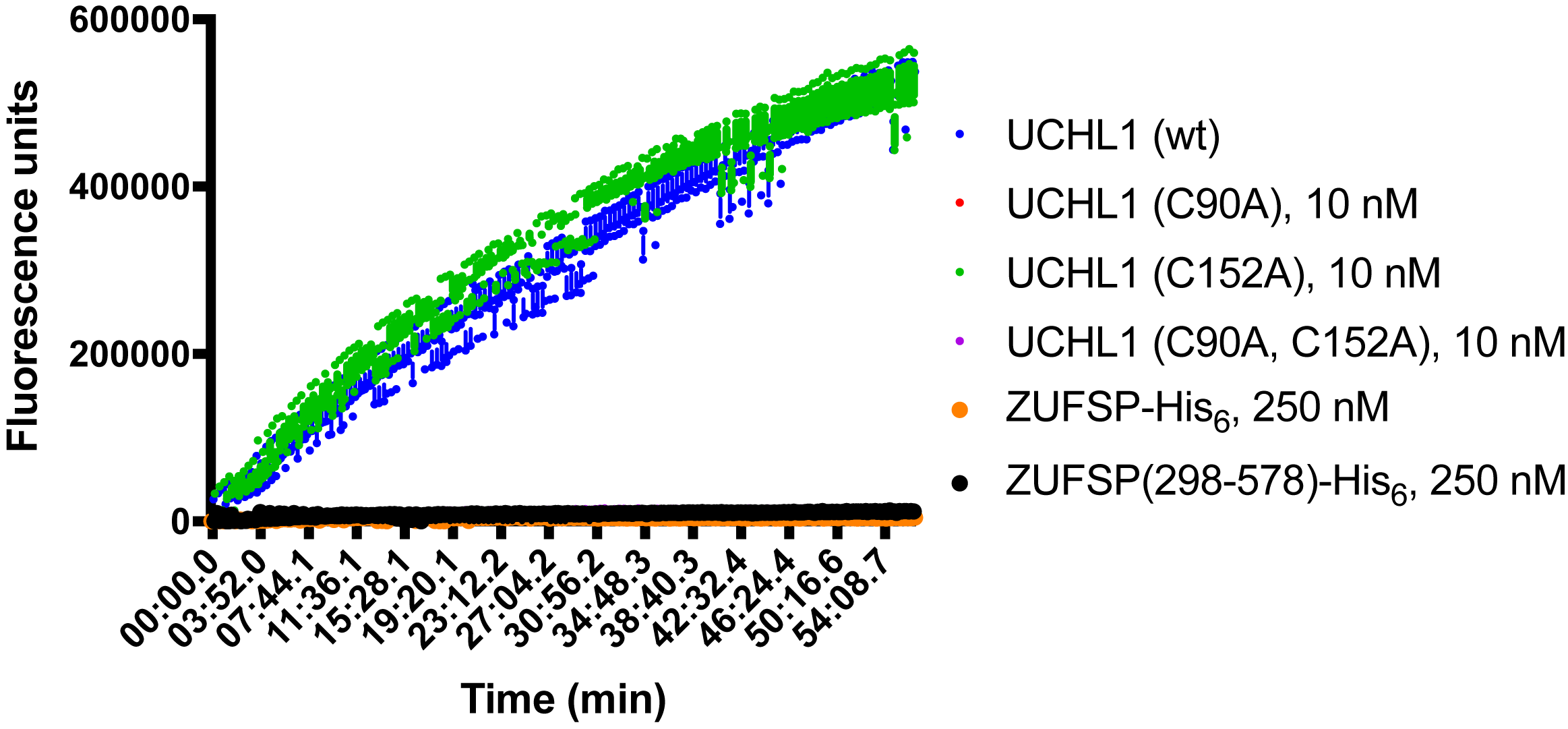


**Supplementary Figure 11 | Hydrolase activity of UCHL1 mutants and ZUFSP.** UCHL1 (10 nM) or ZUFSP (250 nM) was incubated with Ub-AMC (250 nM). The data are arbitrary fluorescence units, presented as the mean of technical triplicates ± SEM. Wild-type and C152A mutants show similar hydrolase activity, while the C152A and C90A,C152A mutants are inactive. ZUSP also shows no hydrolytic activity towards UbAMC.


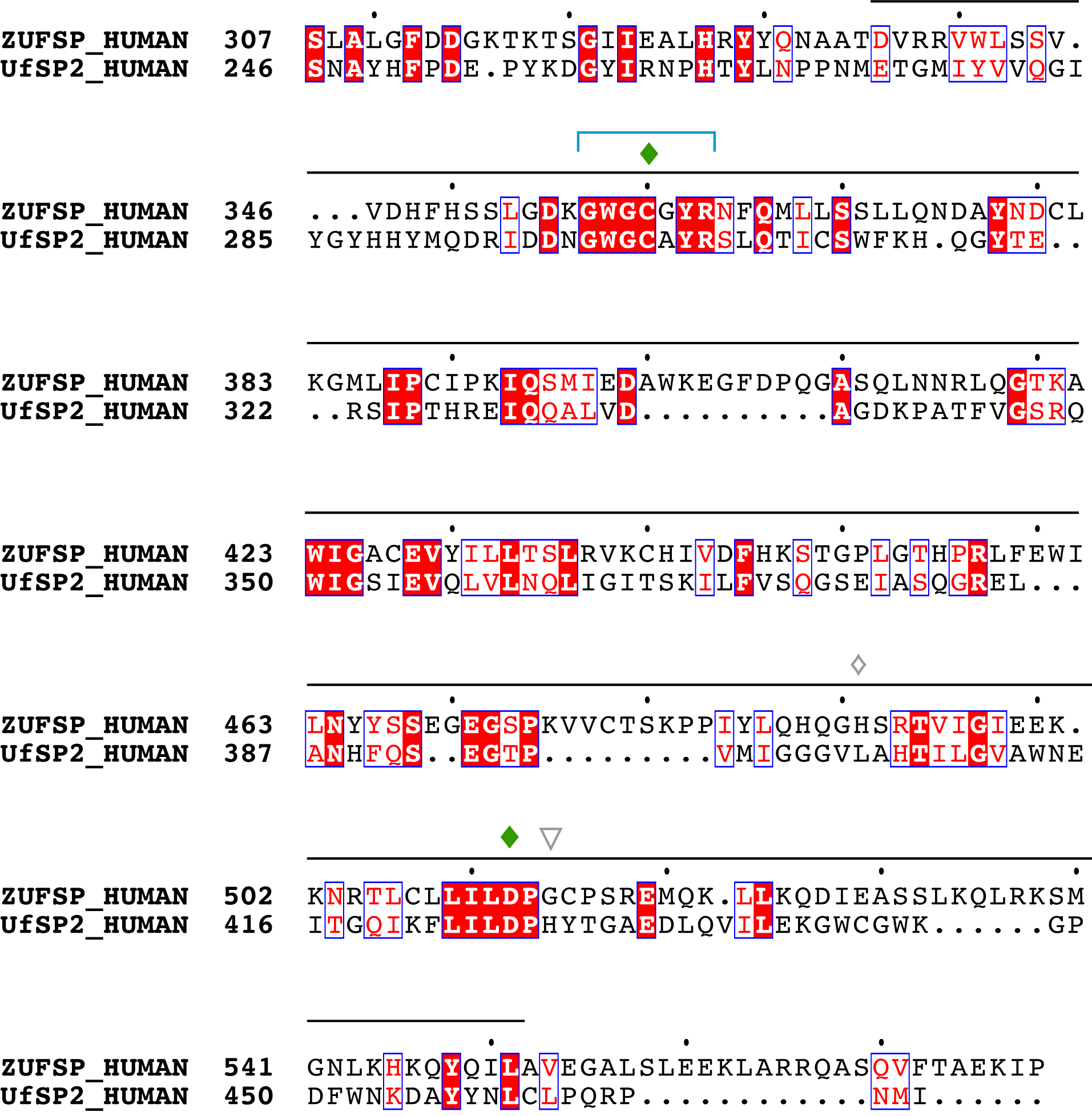


**Supplementary Figure 12 | Alignment of the catalytic domains of human ZUFSP and UfSP2**. Predicted C78 peptidase domain indicated with black line. Labeled peptide from ZUFSP indicated by blue bracket. Catalytic Cys and Asp residues indicated with green diamond; catalytic His from UfSP2 (H428, lacking in ZUFSP) indicated with gray triangle, putative catalytic His (H491) from ZUFSP indicated with gray diamond. Figure produced using ENDscript 2.0 (<http://endscript.ibcp.fr>)^2^.


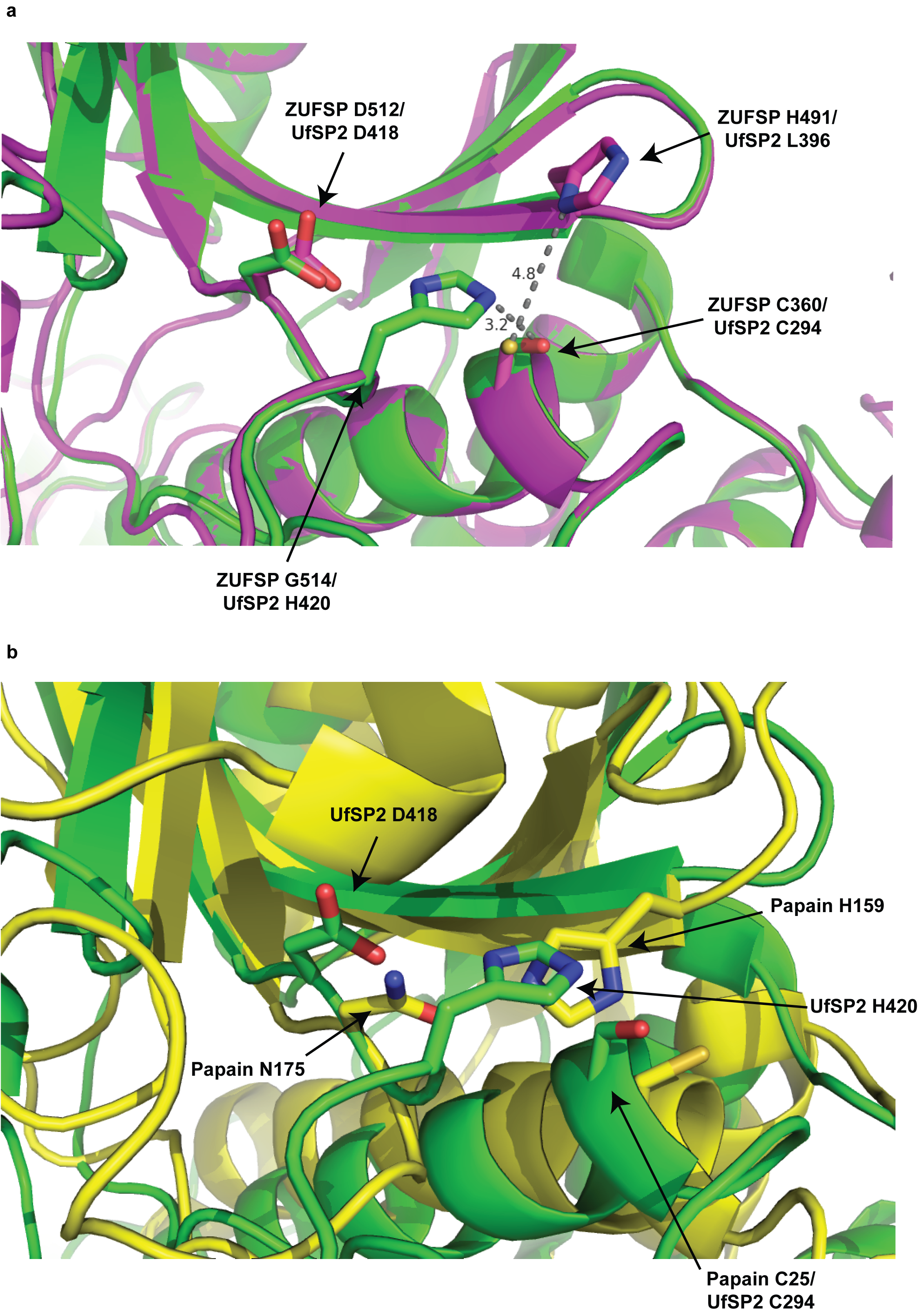


**Supplementary Figure 13 | Comparison of UfSP2 structure with ZUFSP homology model and papain structure.** (**a**) Overlay of ZUFSP homology model (magenta) and mouse UfSP2 structure (green, PBD ID: 3OQC). Model built in SwissModel (<https://swissmodel.expasy.org>) using default parameters based on structure of mouse UfSP2 (PDB ID: 3OQC). (**b**) Overlay of mouse UfSP2 structure (green, PBD ID: 3OQC), and papain (yellow, PDB ID 1PPN).


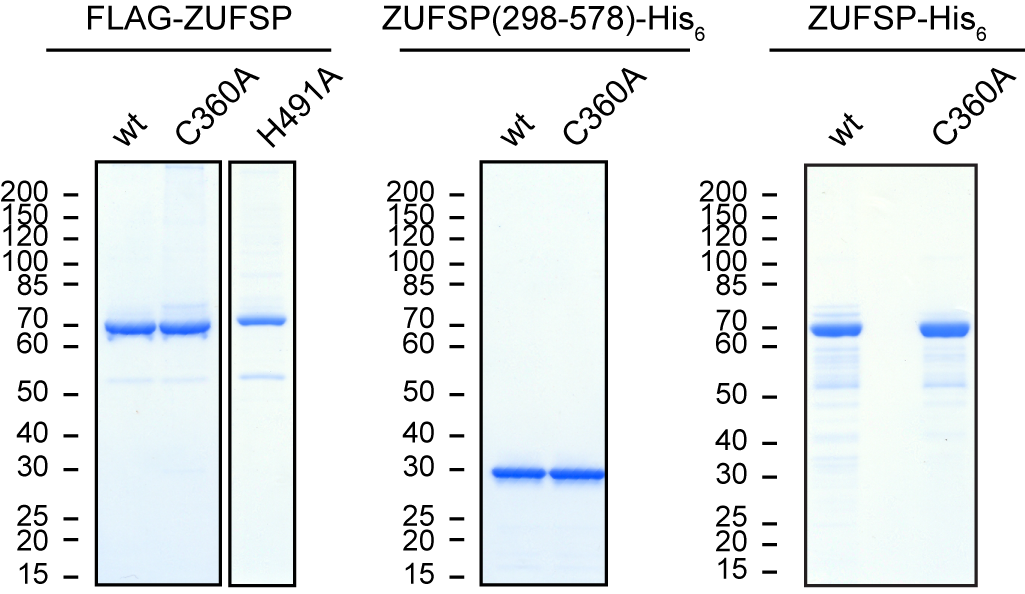


**Supplementary Figure 14 | Coomassie-stained gels of purified ZUFSP**. ZUFSP (2 µg per lane) was run on a 4-12% Bis-Tris gel with MOPS running buffer. Protein was detected with SimplyBlue SafeStain (Invitrogen).


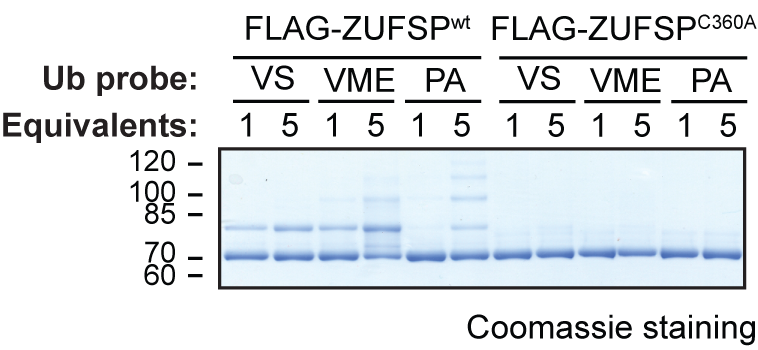


**Supplementary Figure 15 | Probe labeling of FLAG-ZUFSP**. ZUFSP was treated with 1 or 5 eq of ABP for 1 h at 25 °C, then analyzed by SDS-PAGE and stained with SimplyBlue SafeStain (Invitrogen).


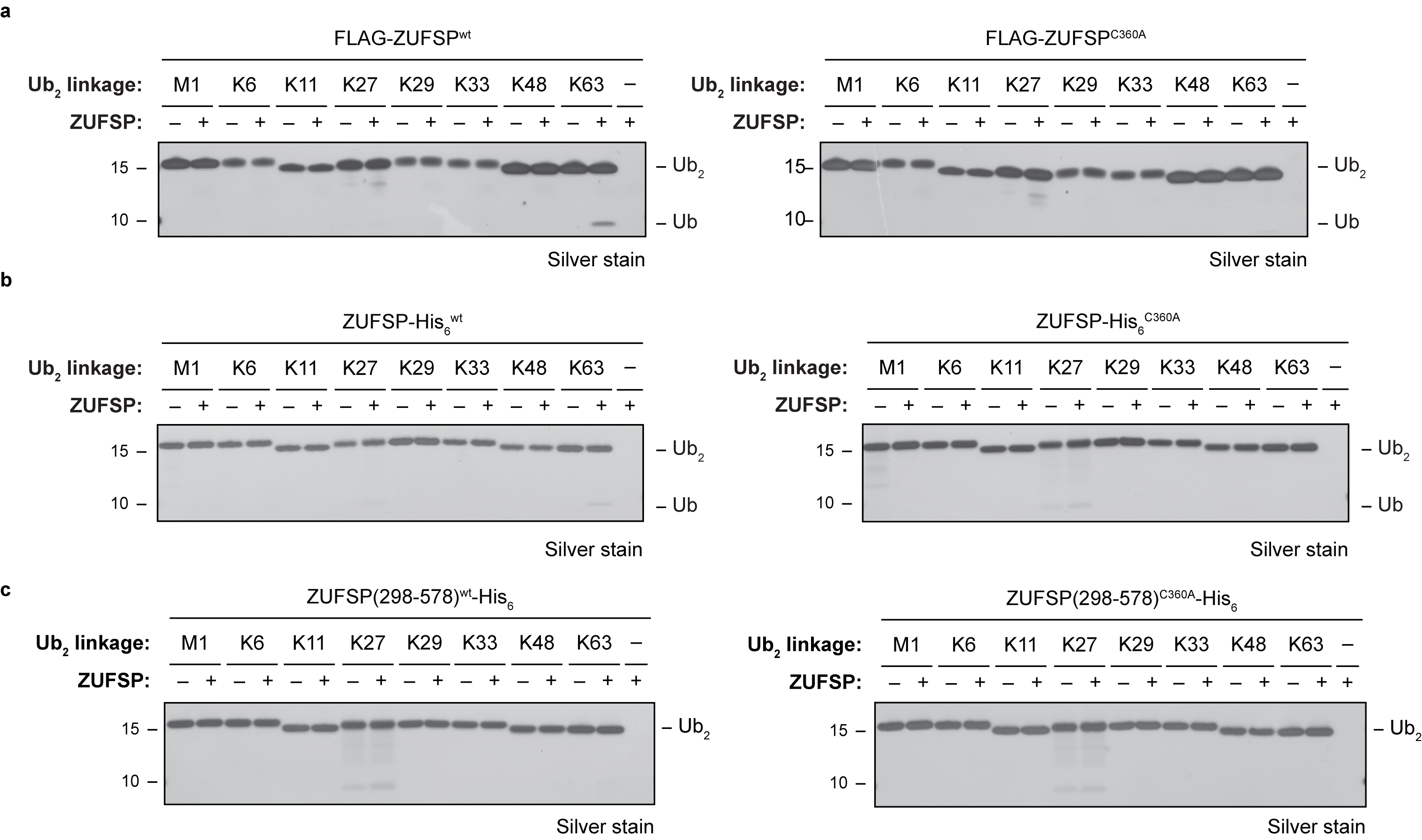


**Supplementary Figure 16 | Di-Ub hydrolysis by ZUFSP.** DUB activity assays testing activity and specificity of ZUFSP towards di- or tetra-ubiquitin chains. 1.1 µM FLAG-ZUFSP (**a**), ZUFSP-His_6_ (**b**) and ZUFSP(298-578)-His_6_ (**c**) was incubated with 2.2 µM Ub chains for 1 h at 25 °C, then analyzed by SDS-PAGE and silver staining.


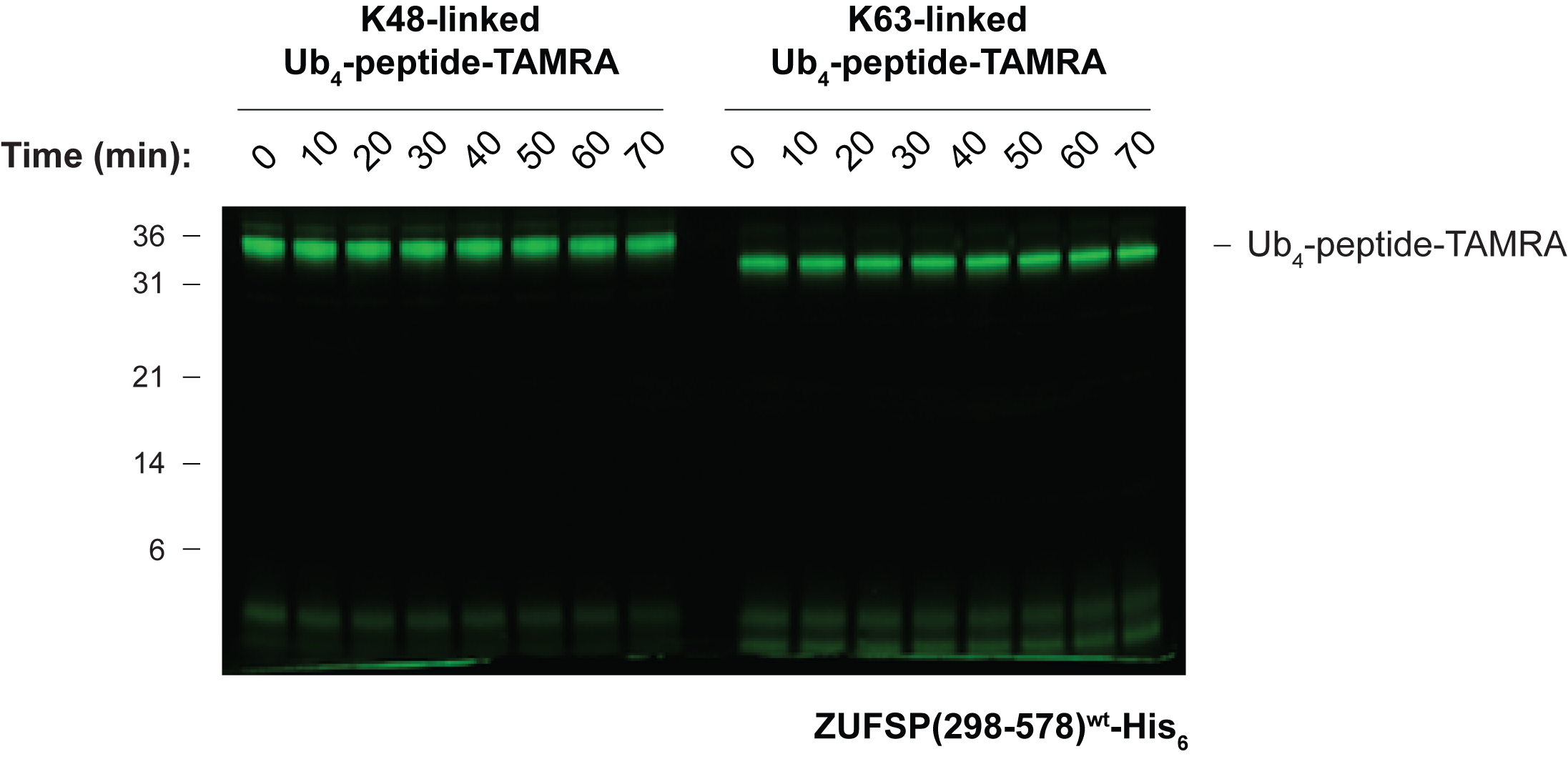


**Supplementary Figure 17 | The catalytic domain of ZUFSP shows no activity towards a tetra-ubiquitin-conjugated peptide.** Time course analysis of ZUFSP(298-578)-mediated depolymerization of K48- and K63-linked tetra-ubiquitin conjugated to a TAMRA-labeled peptide, monitored by in-gel fluorescence. Peptide is at 2.7 µM, ZUFSP-His_6_ is at 1 µM. Figure is representative of two independent replicates.


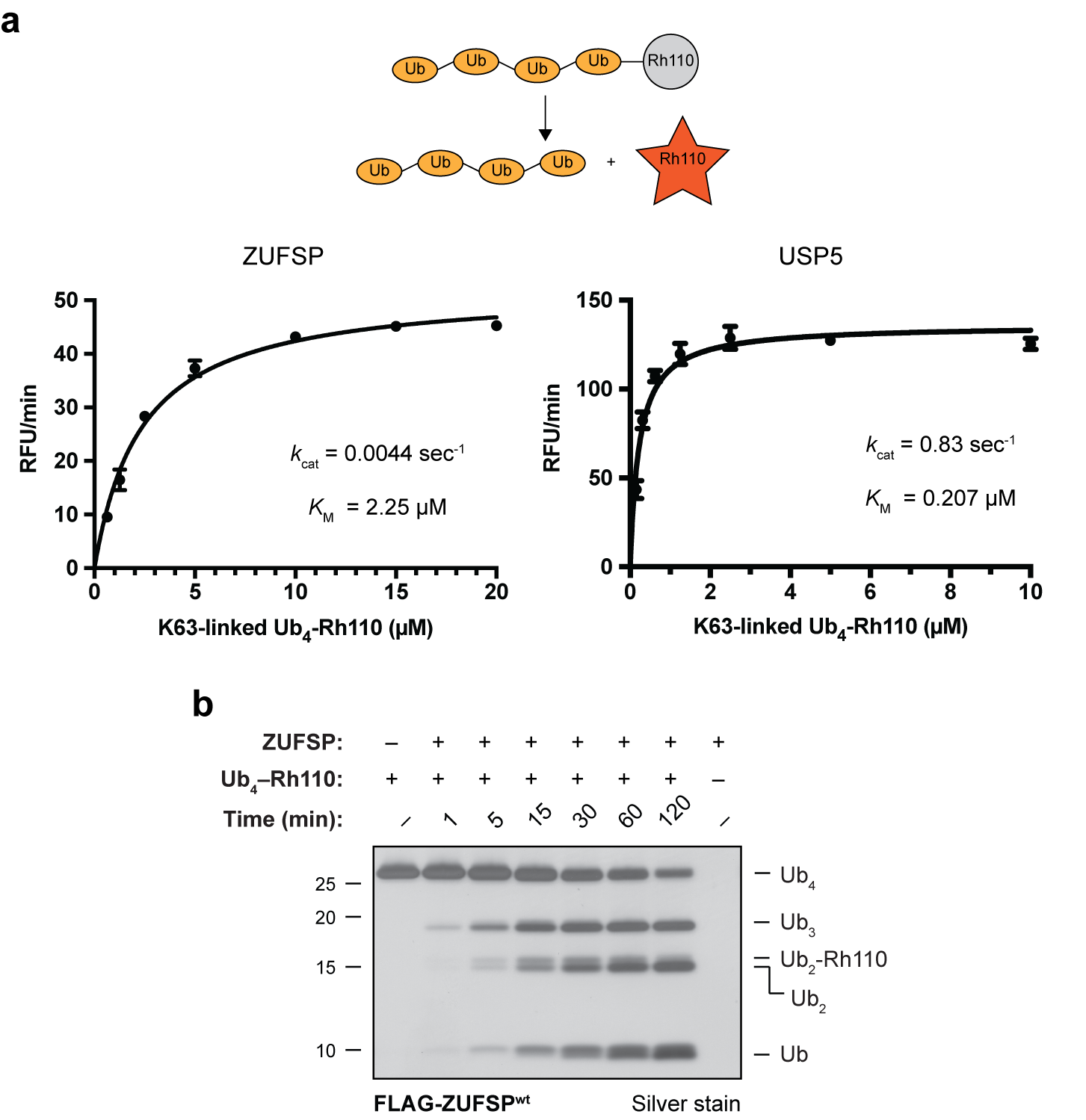


**Supplementary Figure 18 |** **Tetra-ubiquitin-Rh110 hydrolysis by ZUFSP and USP5.** (**a**) ZUFSP-His_6_ (200 nM) or USP5 (5 nM) was incubated with 625 nM – 25 μM 625 nM K63-linked tetra-ubiquitin-Rh110 (for ZUFSP) or 156 nM–10 µM (for USP5). The increase in fluorescence at early time points from free Rh110 was used to obtain initial velocities (*V*_0_). Values are the mean of three biological replicates. Note that this method only detects the hydrolysis of the proximal Ub-fluorophore bond, not internal sites. Since biphasic kinetics are not observed, it is likely that at early time points the measured rates reflect hydrolysis at the proximal end of the tetra-Ub chain, not hydrolysis of smaller Ub-Rh110 conjugates (or that these substrates are processed at similar rates). (**b**) Ub_4_-Rh110 (2.2 µM) cleavage by FLAG-ZUSP (1.1 µM).

**Supplementary Figure 19 | Effect of ubiquitin chains on hydrolysis of K63-linked Ub4_4_-Rh110 by ZUFSP.** The initial rate of the hydrolysis of the fluorogenic ZUFSP substrate K63-linked Ub4_4_-Rh110 was measured in the presence of varying concentrations of Ub chains or free Ub.


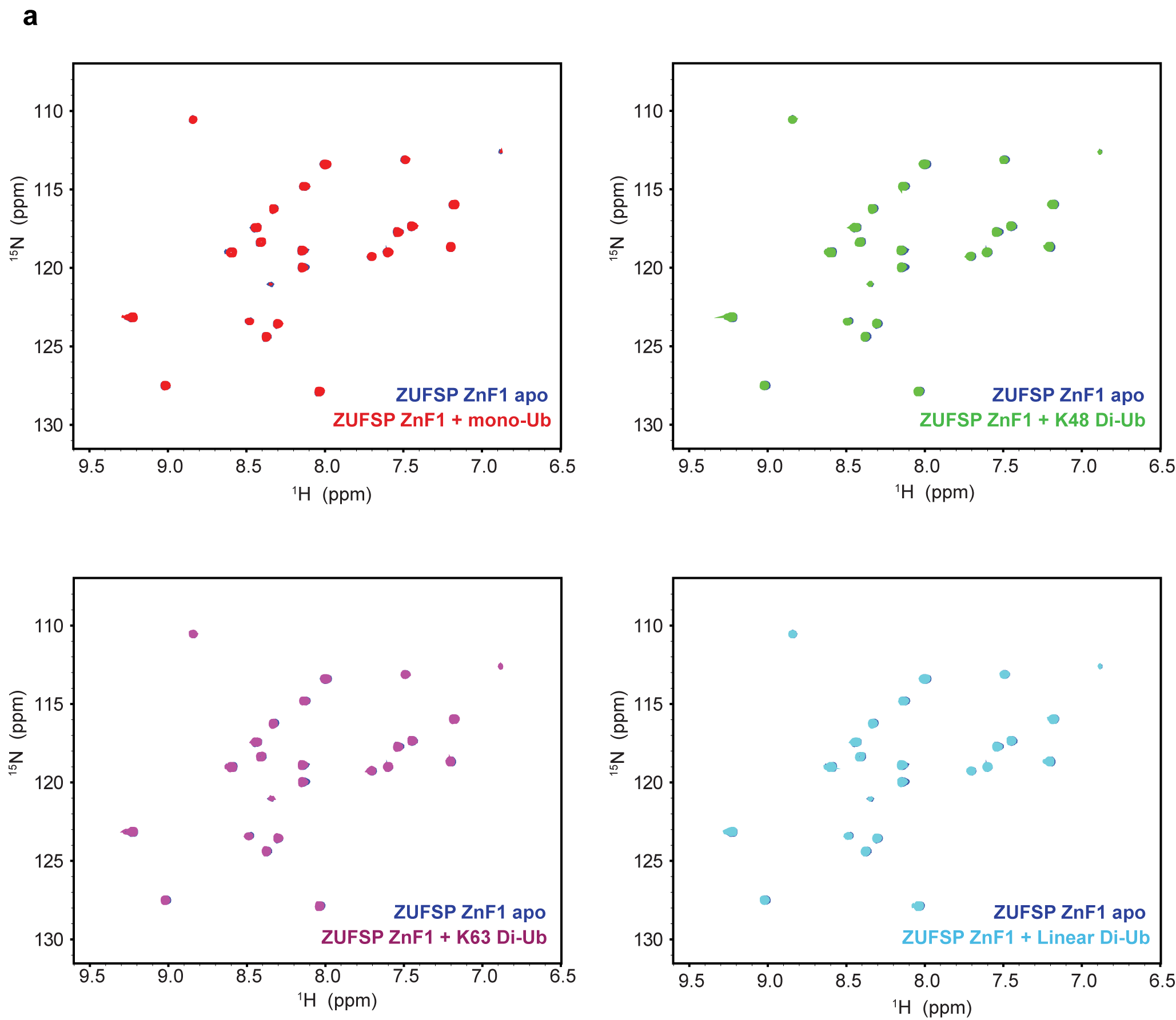


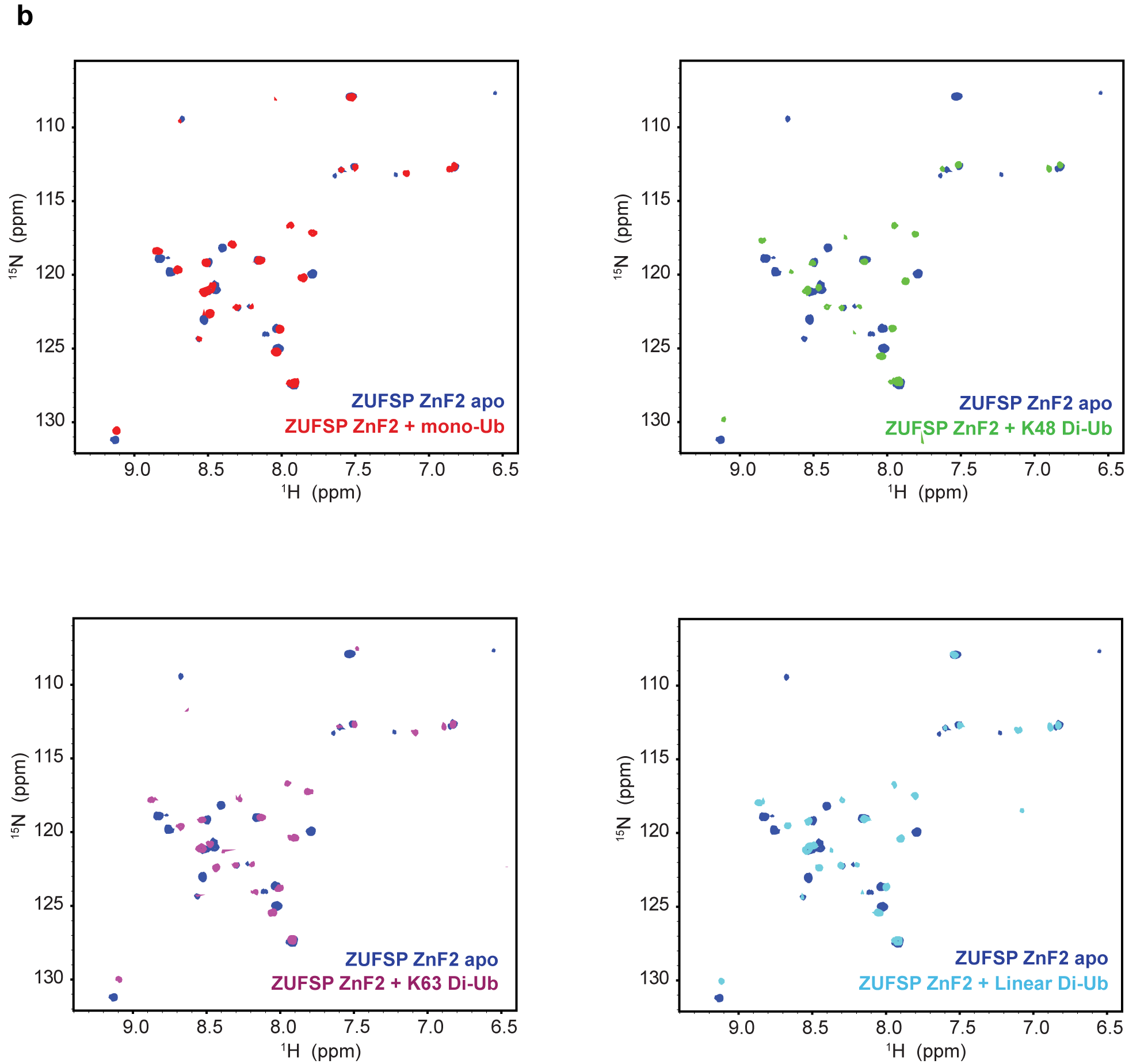


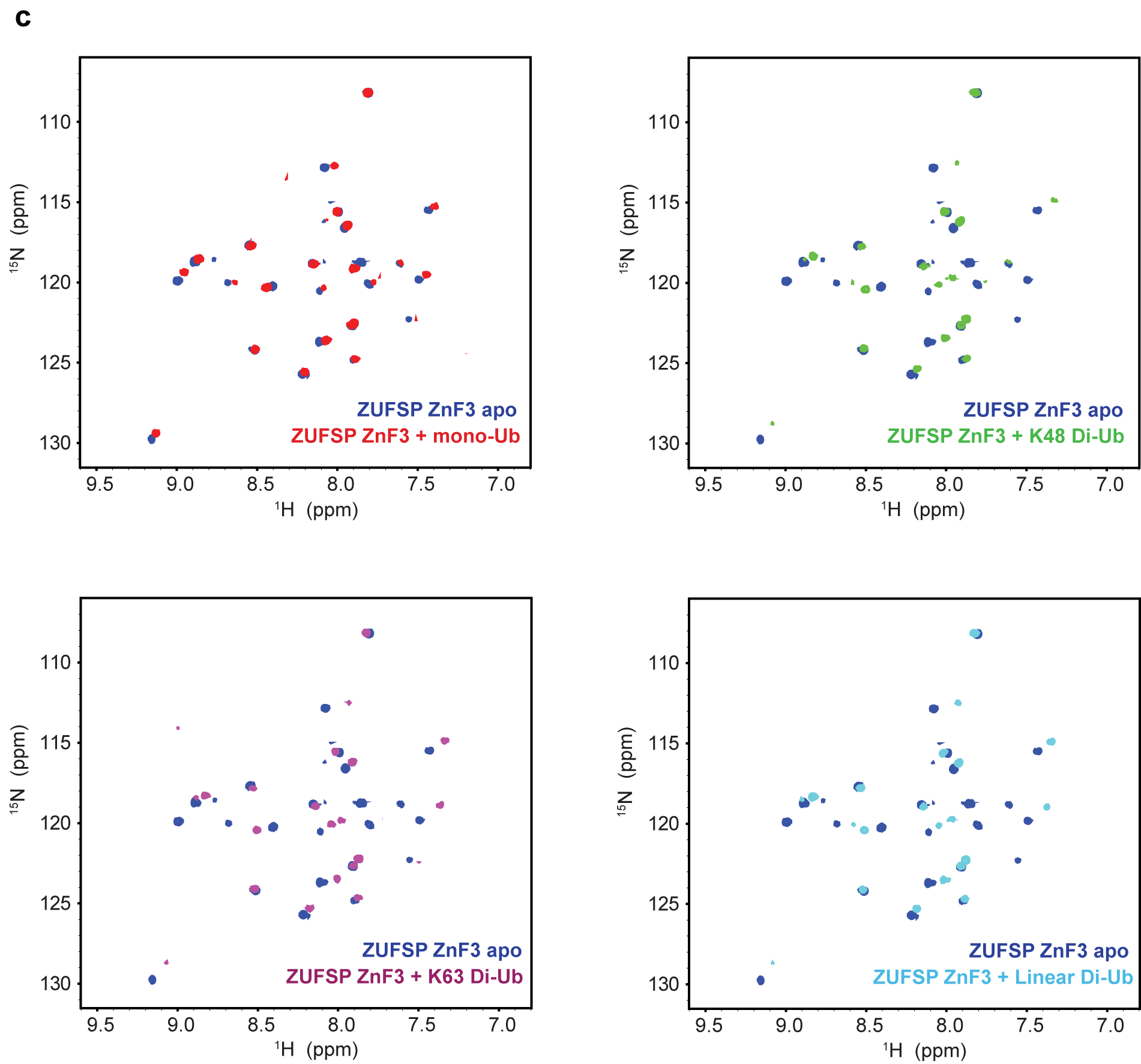


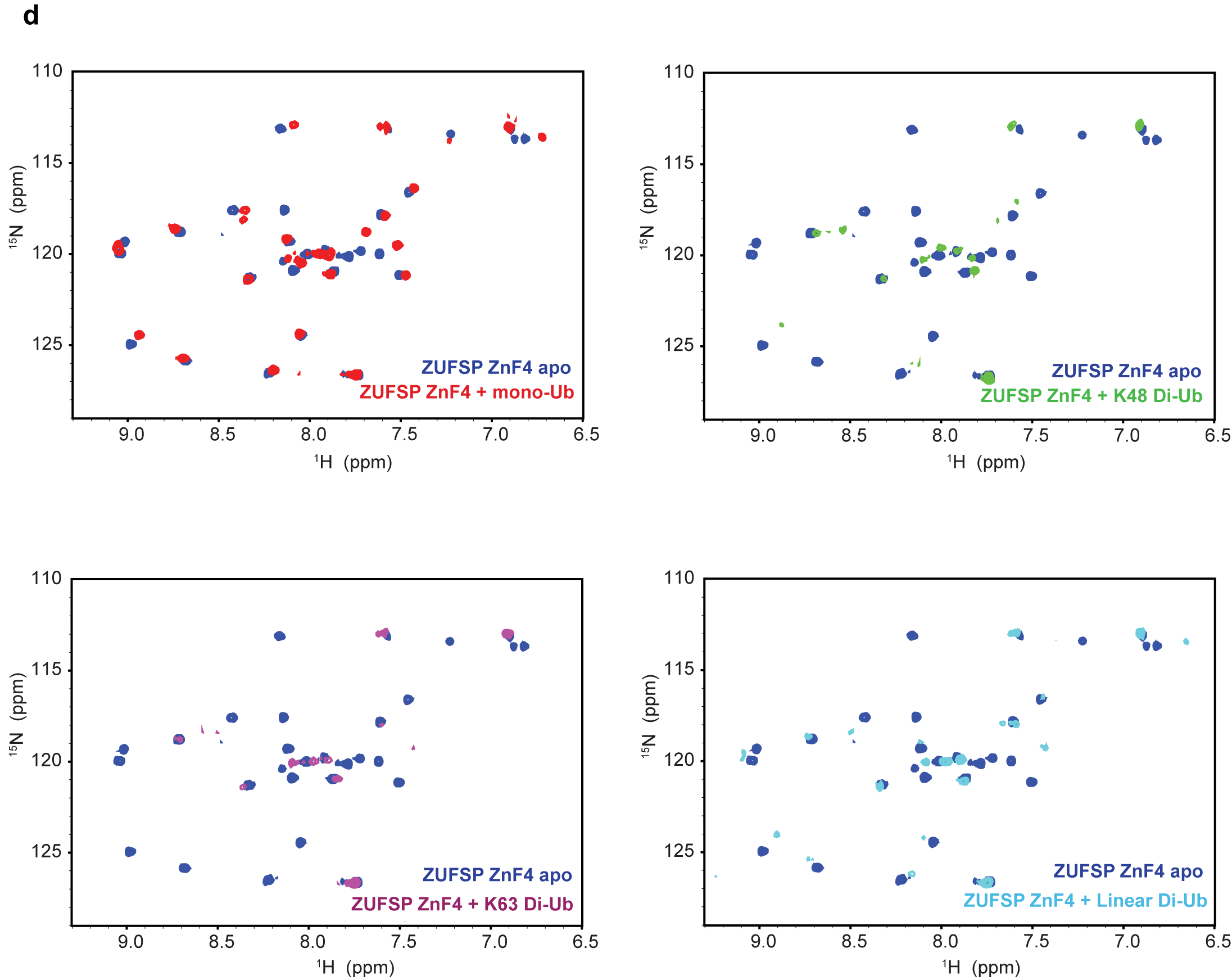


**Supplementary Figure 20 | Binding of ZUFSP ZnF domains to different ubiquitin linkages.** Overlaid ^1^H-^15^N HSQC spectra of ^15^N labeled apo ZnFs (blue) bound to unlabeled mono-Ubiquitin (red), K48-linked di-Ubiquitin chain (green), K63-linked di-Ubiquitin chain (magenta) and linear di-Ubiquitin chain (cyan) in 1:1 ratio. Binding to all ubiquitin linkages were observed in ZnF2 (**b**), ZnF3 (**c**) and ZnF4 (**d**); however, no binding was observed for any of the linkages with ZnF1 (**a**).


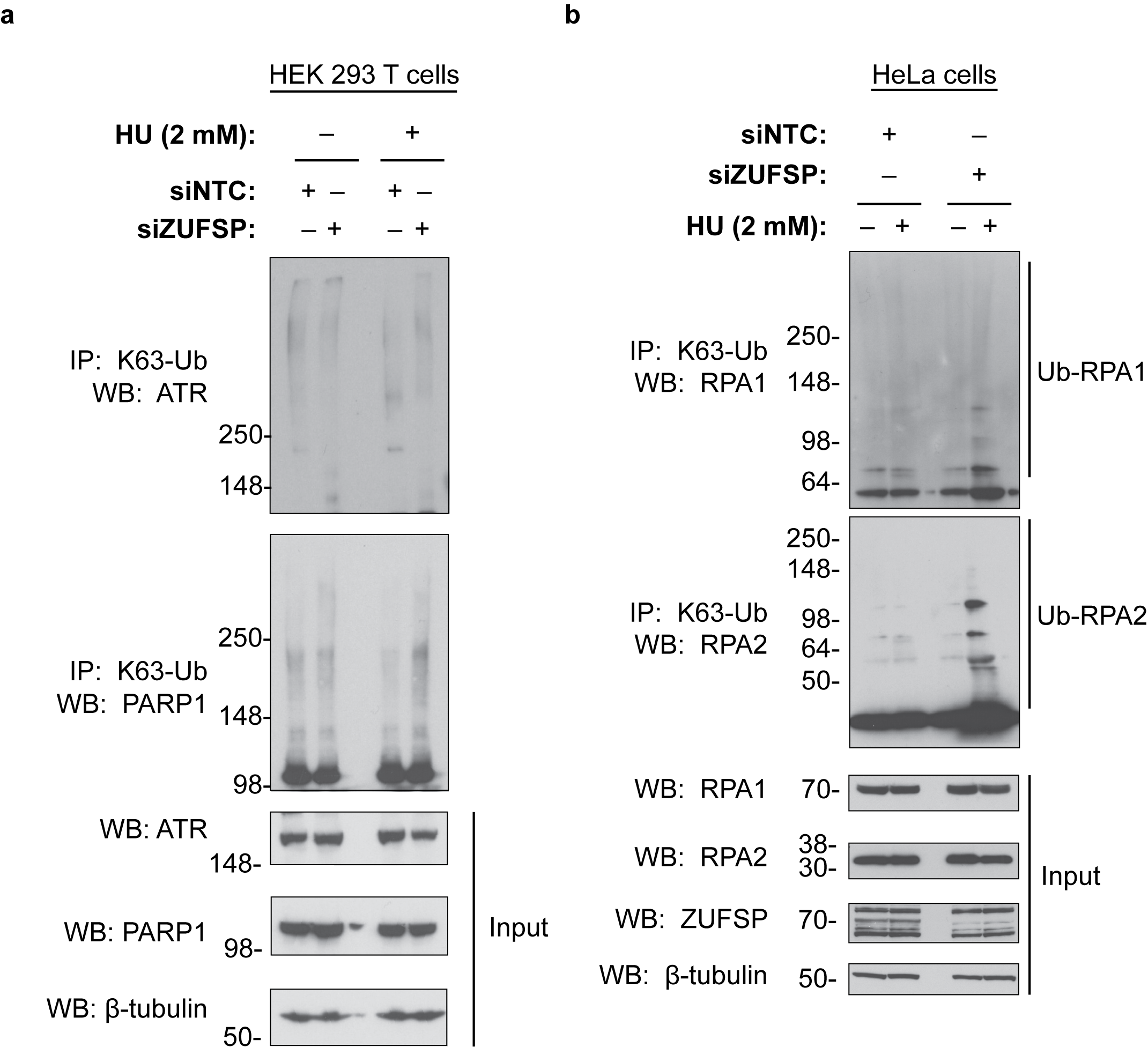


**Supplementary Figure 21 | Additional Ub-K63 immuoprecipitations.** (**a**) Western blots for ATR and PARP1 after Ub-K63 immunoprecipitation in HEK 293T cells, following ZUFSP knock-down and HU treatment. (**b**) Western blots for RPA1 and RPA2 after Ub-K63 immunoprecipitation in HeLa cells, following ZUFSP knock-down and HU treatment.


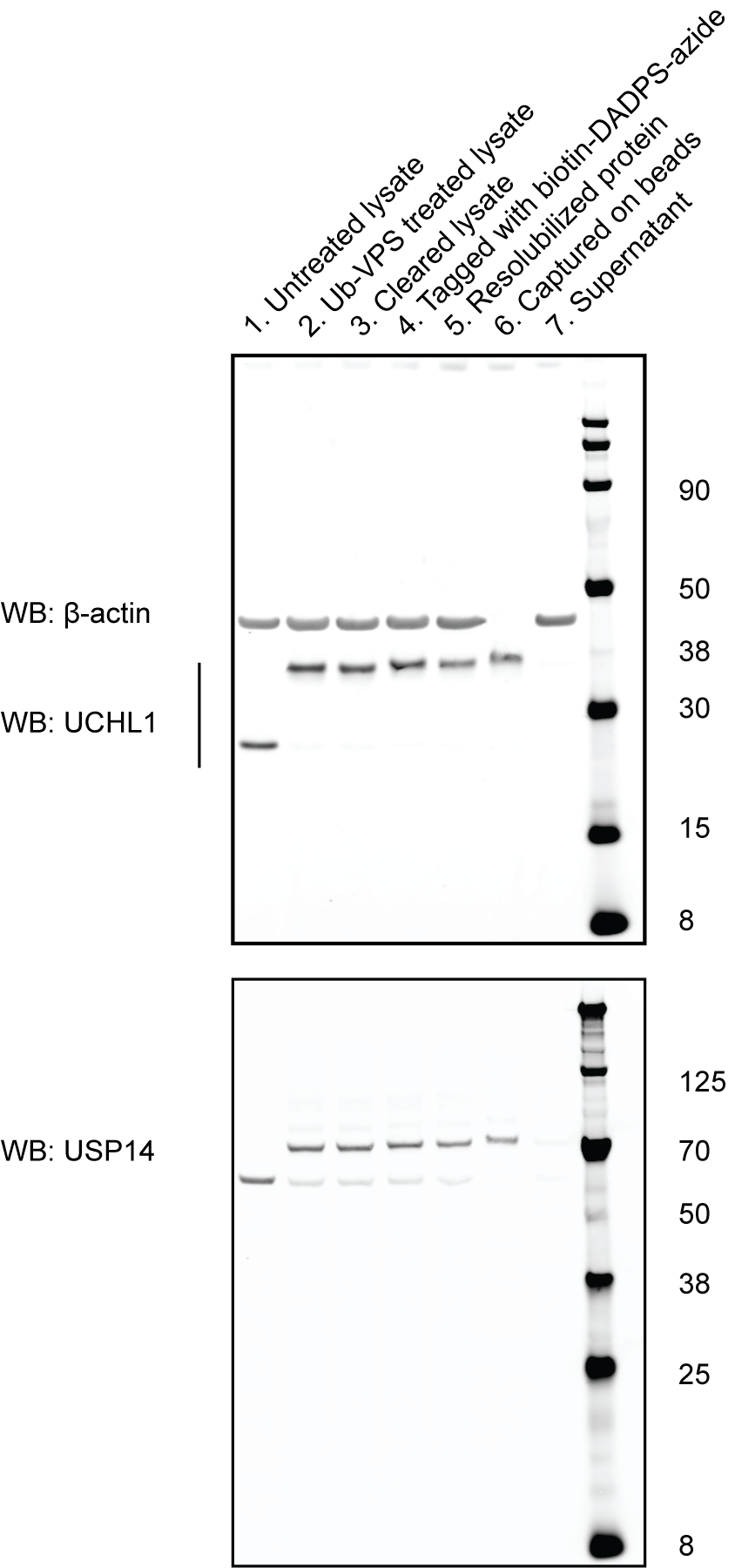


**Supplementary Figure 22.** Uncropped gel from Figure 2.


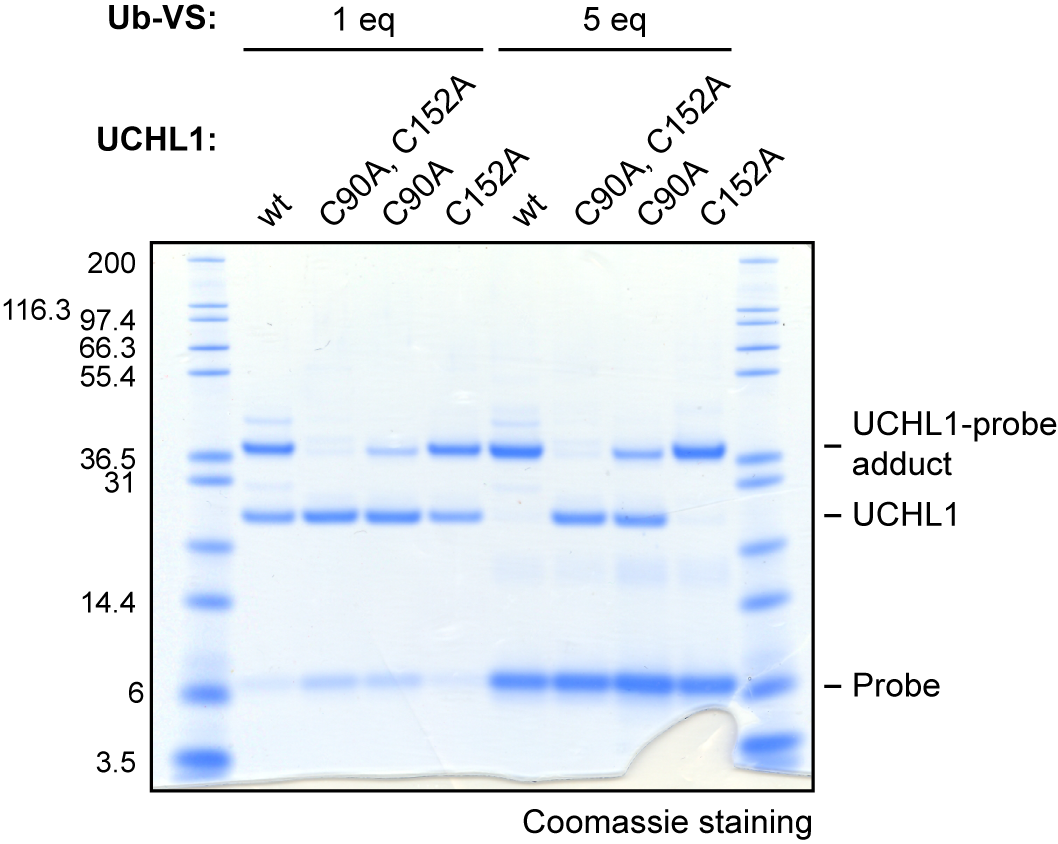


**Supplementary Figure 23.** Uncropped gel from Figure 4.


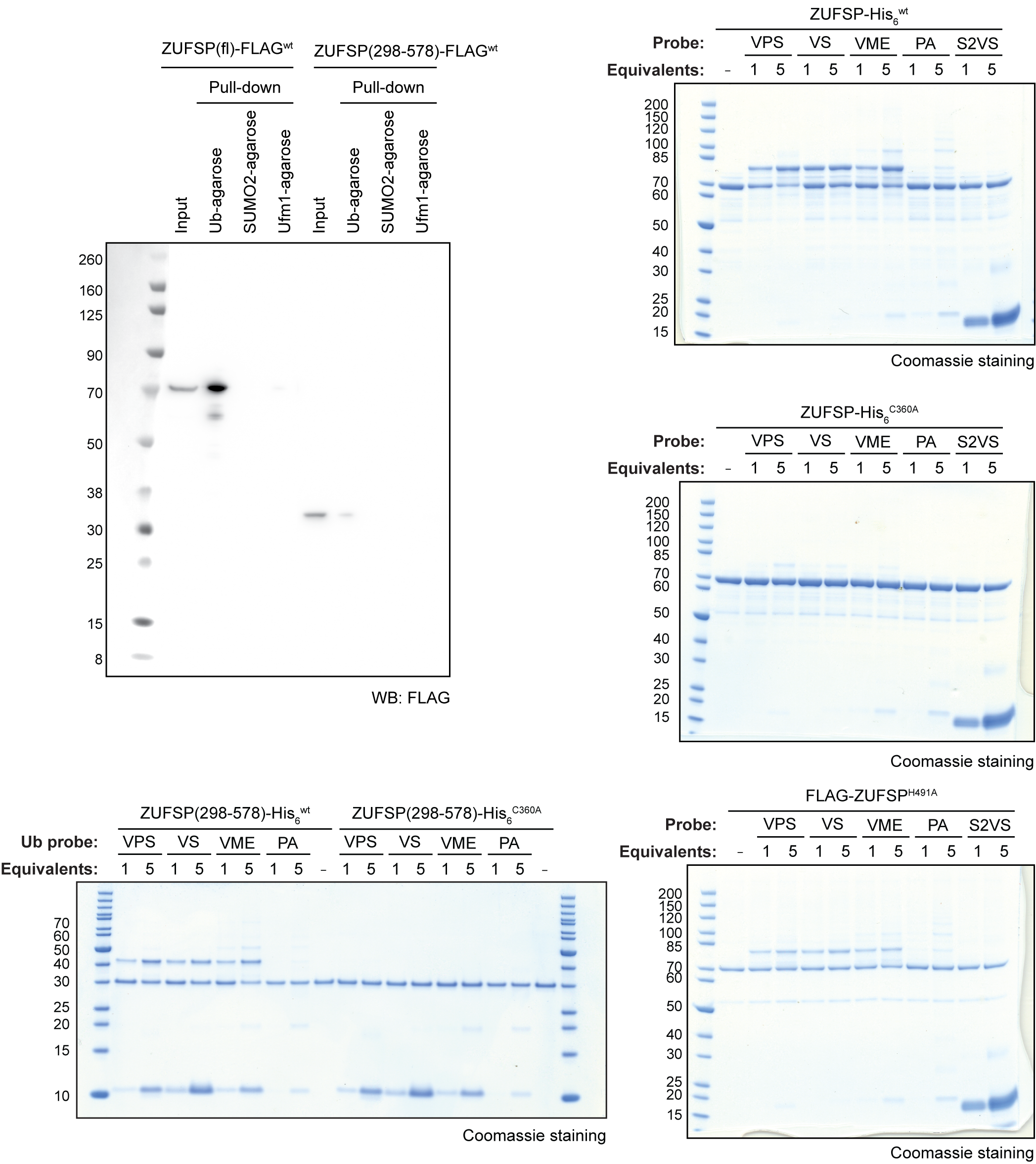


**Supplementary Figure 24.** Uncropped blots/gels from Figure 5.


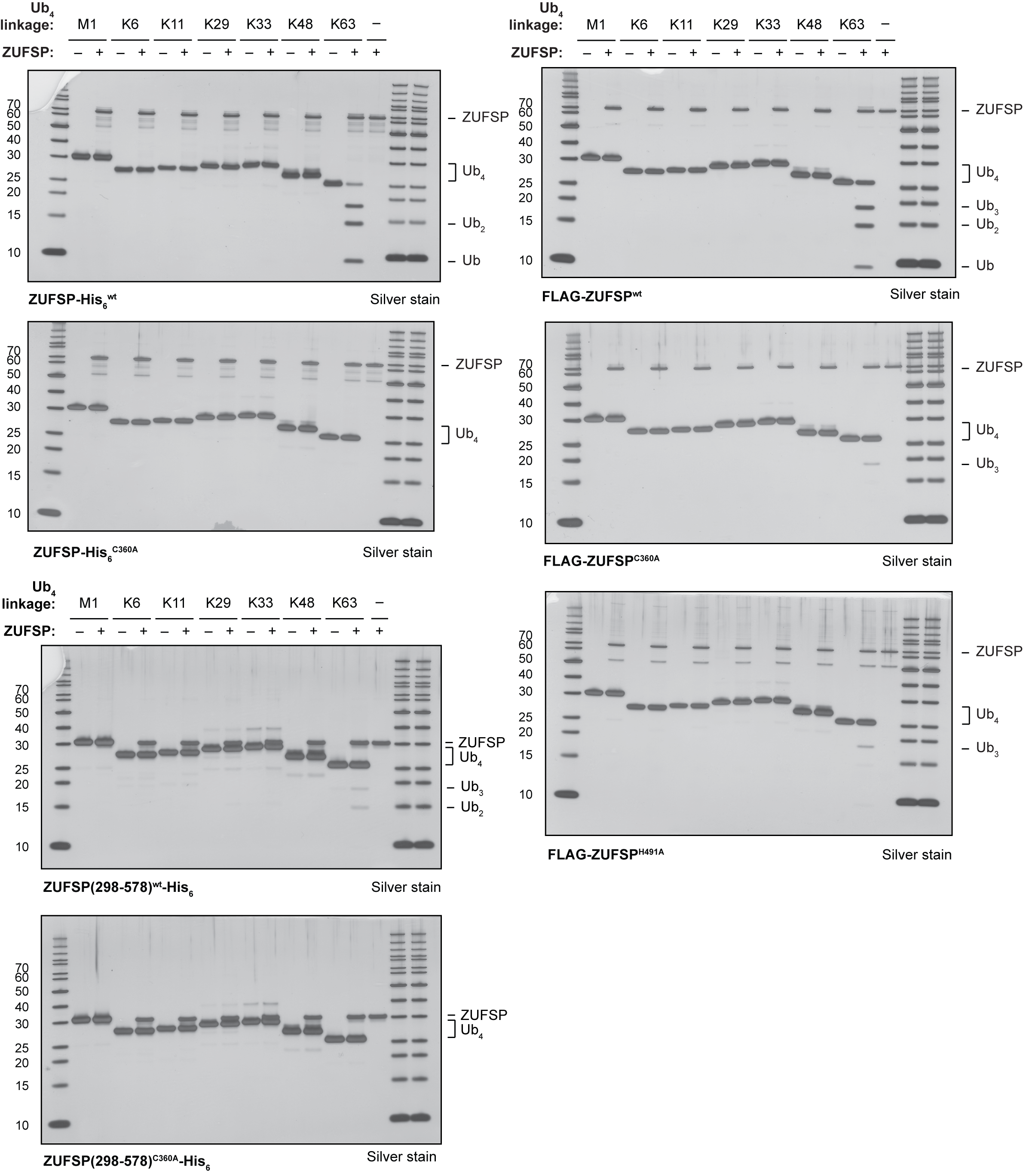


**Supplementary Figure 25.** Uncropped gels from Figure 6.


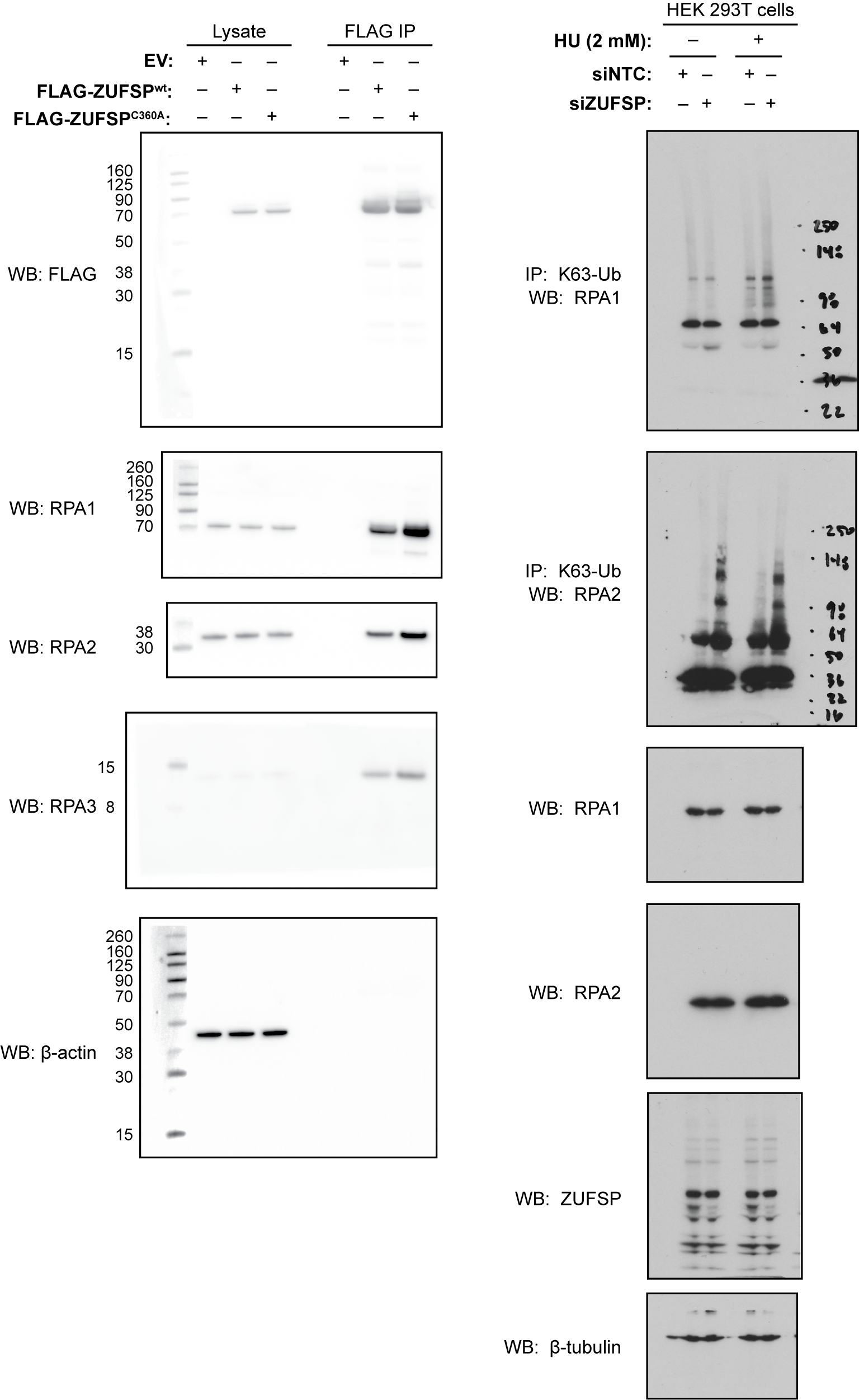


**Supplementary Figure 26.** Uncropped blots from Figure 7.

**Supplementary Methods**

**Small-molecule synthesis**

General methods

All solvents and reagents were used as obtained. NMR analysis was performed in a deuterated solvent with a Bruker Avance 400 MHz NMR spectrometer, referenced to trimethylsilane (TMS). Chemical shifts are expressed as δ units using TMS as the internal standard (in NMR description, s = singlet, d = doublet, t = triplet, q = quartet, m = multiplet, and br = broad peak). All coupling constants (*J*) are reported in Hertz. Mass spectra were measured with a Finnigan SSQ710C spectrometer using an ESI source coupled to a Waters 600MS high performance liquid chromatography (HPLC) system operating in reverse-phase mode with an X-bridge Phenyl column of dimensions 150 mm by 2.6 mm, with 5 μm sized particles. Preparatory-scale silica gel chromatography was performed using medium-pressure liquid chromatography (MPLC) on a CombiFlash Companion (Teledyne ISCO) with RediSep normal phase silica gel (35−60 *μ*m) columns and UV detection at 254 or 220 nm. Thin-layer chromatography was performed on glass-backed silica plates with visualization under UV (254 nm) or staining with basic potassium permanganate.

*tert*-Butyl (2-oxoethyl)carbamate **1**^3^

*tert*-Butyl *N*-(2,3-dihydroxypropyl)carbamate (2.00 g, 10.5 mmol, 1.0 eq) was dissolved in H_2_O (17 mL) and NaIO_4_ (2.50 g, 12.0 mmol, 1.1 eq) was added. The reaction was stirred in the dark for 3 h, then a colorless precipitate was filtered off. The aqueous solution was extracted with CHCl_3_ (6 × 15 mL). The combined organic layers were dried over MgSO_4_, filtered, and concentrated *in vacuo* to give **1** as a colorless oil which solidified to a waxy colorless solid upon storage at –20 ºC (1.57 g, 94%); ^1^H NMR (400 MHz, Chloroform-d) δ 9.66 (s, 1H), 5.17 (br s, 1H), 4.08 (d, *J* = 5.1 Hz, 2H), 1.46 (s, 9H). The data are in good agreement with literature values.

*S*-(Pent-4-yn-1-yl) ethanethioate **2**^4^

Following the procedure of Benati *et al*.,^4^ potassium thioacetate (2.8 g, 24 mmol) was added to a solution of 5-chloropent-1-yne (2.00 g, 19.5 mmol, 1.0 eq) in acetone (50 mL). The suspension was refluxed for 7 h, then cooled to rt and stirred at rt for 3 days. The resultant orange-brown suspension was filtered through Celite (eluent acetone) and concentrated *in vacuo*. The residue was purified through a plug of silica, loading in heptanes and eluting with 30% CH_2_Cl_2_/heptanes, to give **2** as a brown oil (2.42 g, 87%); R_f_ 0.3 (30% CH_2_Cl_2_/heptanes); ^1^H NMR (400 MHz, CDCl_3_) ^1^H NMR (400 MHz, Chloroform-*d*) δ 2.99 (t, *J* = 7.0 Hz, 2H), 2.33 (s, 3H), 2.28 (td, *J* = 7.0, 2.7 Hz, 2H), 1.97 (t, *J* = 2.7 Hz, 1H), 1.81 (quintet, *J* = 7.0 Hz, 2H); LRMS (ES^+^) *m*/*z* 143 ([M+H]^+^). The data are in good agreement with literature values.

Pent-4-yne-1-thiol **3**^4^

Following the procedure of Benati *et al*.,^4^ **2** (2.4 g, 17 mmol) was dissolved in anhydrous MeOH (60 mL) and sodium ethanethiolate (1.2 g, 17 mmol. 1.0 eq) was added. The reaction was stirred under a nitrogen atmosphere at rt for 1 h, after which time TLC analysis indicated complete consumption of thioester. The reaction was quenched with 200 mL 0.2 M aq. HCl and extracted with CH_2_Cl_2_ (3 × 75 mL). The combined organic layers were washed with brine (200 mL), dried (MgSO_4_), and concentrated *in vacuo* to give **3** as a brown oil which was used immediately without further purification (1.49 g, 88%); ^1^H NMR (400 MHz, CDCl_3_) ^1^H NMR (400 MHz, Chloroform-*d*) δ 2.70 – 2.63 (m, 2H), 2.35 (td, *J* = 6.8, 2.7 Hz, 2H), 1.96 (t, *J* = 2.7 Hz, 1H), 1.87 – 1.77 (m, 3H), 1.35 (t, *J* = 8.1 Hz, 1H). The data are in good agreement with literature values.

Diethyl ((pent-4-yn-1-ylthio)methyl)phosphonate **4**

Following the procedure of Wang and Yao,^5^ to a dry flask under nitrogen were added sequentially diethyl iodomethylphosphonate (3.27 g, 11.8 mmol, 1.0 eq), anhydrous DMF (45 mL), **3** (1.3 g, 13 mmol, 1.1 eq) and Cs_2_CO_3_ (7.9 g, 24 mmol, 2.1 eq). The suspension was stirred at rt for 18 h, then quenched with aq. HCl (5 M, 20 mL), diluted with water (100 mL) and extracted with EtOAc (4 × 100 mL). The organic extracts were washed with aq. HCl (0.1 M, 200 mL) and brine (200 mL), then dried (MgSO_4_), filtered and concentrated *in vacuo*. Purification by silica gel column chromatography (eluent 10-100% *^i^*PrOAc/heptanes) gave **4** as a brown oil (2.67 g, 91%); R_f_ 0.31 (50% EtOAc/heptanes); ^1^H NMR (400 MHz, Chloroform-*d*) δ 4.27 – 4.09 (m, 4H), 2.84 (td, *J* = 7.1, 1.0 Hz, 2H), 2.73 (d, *J* = 13.4 Hz, 2H), 2.33 (td, *J* = 7.1, 2.7 Hz, 2H), 1.97 (t, *J* = 2.7 Hz, 1H), 1.84 (quin, *J* = 7.0 Hz, 2H), 1.35 (t, *J* = 7.1 Hz, 6H); ^13^C NMR (101 MHz, Chloroform-*d*) δ 83.3, 69.1, 62.7 (d, *J* = 7.0 Hz), 32.4 (d, *J* = 3.4 Hz), 27.8, 25.3 (d, *J* = 150.6 Hz), 17.4, 16.5 (d, *J* = 6.0 Hz); ^31^P NMR (162 MHz, Chloroform-*d*) δ 24.2; LRMS (ES^+^) *m*/*z* 251 ([M+H]^+^), 501 ([2M+H]^+^).

Diethyl ((pent-4-yn-1-ylsulfonyl)methyl)phosphonate **5**

To a solution of **4** (1.34 g, 5.35 mmol, 1.0 eq) in MeOH (15 mL) at 0 ºC was added a suspension of Oxone (4.94 g, 8.03 mmol, 1.5 eq) in H_2_O (21 mL). The suspension was stirred at 0 ºC for 1 h then at rt for 6 h, after which time TLC analysis indicated complete consumption of starting material. The suspension was filtered through Celite and concentrated *in vacuo* to remove MeOH. The remaining cloudy aqueous suspension was extracted with EtOAc (3 × 25 mL). The combined organic extracts were washed with brine (75 mL), then dried (MgSO_4_), filtered and concentrated *in vacuo*. Purification by silica gel column chromatography (eluent 25-100% EtOAc/heptane) gave **5** as a pale yellow oil (1.08 g, 72%); R_f_ 0.52 (EtOAc); ^1^H NMR (400 MHz, Chloroform-d) δ 4.29 – 4.19 (m, 4H), 3.57 (d, *J* = 16.5 Hz, 2H), 3.52 – 3.45 (m, 2H), 2.42 (td, *J* = 6.8, 2.6 Hz, 2H), 2.16 – 2.05 (m, 2H), 2.04 (t, *J* = 2.6 Hz, 1H), 1.38 (t, *J* = 7.1 Hz, 6H); ^13^C NMR (101 MHz, Chloroform-*d*) δ 81.8, 70.3, 63.6 (d, *J* = 6.5 Hz), 53.3, 50.60 (d, *J* = 138.7 Hz), 20.9, 17.4, 16.3 (d, J = 6.0 Hz); ^31^P NMR (162 MHz, Chloroform-*d*) δ 11.8; LRMS *m*/*z* (ES^+^) 283 ([M+H]^+^), 300 ([M+NH_4_]^+^)’ HRMS (m/z): [M]^+^ calcd. for C_10_H_20_O_4_PS, 283.0764; found, 283.0751.

*tert*-Butyl (*E*)-(3-(pent-4-yn-1-ylsulfonyl)allyl)carbamate **6**

Following the procedure of Claridge *et al.*^6^, MeMgBr (3.0 M in Et_2_O, 1.1 mL, 3.3 mmol, 1.0 eq) was added dropwise to a solution of **5** (900 mg, 3.19 mmol, 1.0 eq) in anhydrous THF (20 mL) at 0 ºC. The solution was warmed to rt and stirred for 15 min. A solution of **1** (508 mg, 3.19 mmol, 1.0 eq) in anhydrous THF (10 mL) was then added dropwise, and the solution was heated at reflux for 2.5 h. The reaction was then cooled to rt, quenched with sat. aq. NH_4_Cl (25 mL) and extracted with Et_2_O (3 × 25 mL). The combined organic layers were washed with brine (75 mL), dried (MgSO_4_), filtered and concentrated *in vacuo* to give **6** as a pale yellow viscous gum which solidified slowly to a beige solid (888 mg, 97%); ^1^H NMR (400 MHz, Chloroform-*d*) δ 6.90 (dt, *J* = 15.2, 4.4 Hz, 1H), 6.43 (dt, *J* = 15.2, 1.9 Hz, 1H), 4.77 (s, 1H), 4.00 (s, 2H), 3.17 – 3.08 (m, 2H), 2.38 (td, *J* = 6.8, 2.6 Hz, 2H), 2.11 – 1.94 (m, 3H), 1.46 (s, 9H); LRMS (ES^+^) 305 ([M+NH_4_]^+^); HRMS (m/z): [M]^+^ calcd. for C_13_H_21_O_4_NaS, 301.1084; found, 301.1070.

(*E*)-3-(Pent-4-yn-1-ylsulfonyl)prop-2-en-1-ammonium trifluoroacetate **7**

A solution of **6** (94 mg) in CH_2_Cl_2_ (1 mL) and TFA (0.5 mL) was stirred for 30 min, after which time TLC analysis indicated complete consumption of starting material. The reaction mixture was concentrated from toluene (3 × 1 mL) and dried *in vacuo*. The brown residue was dissolved in approximately 1.5 mL MeOH and dropped into 25 mL ice-cold Et_2_O. The resultant precipitate was filtered, washed with Et_2_O and dried *in vacuo* to give **7** as fine beige needles (76 mg, 77%); ^1^H NMR (400 MHz, DMSO-*d*_6_) δ 8.36 (s, 3H), 6.93 (d, *J* = 15.4 Hz, 1H), 6.76 (dt, *J* = 15.4, 5.3 Hz, 1H), 3.81 – 3.74 (m, 2H), 3.22 – 3.13 (m, 2H), 2.88 (t, *J* = 2.7 Hz, 1H), 2.32 (td, *J* = 7.2, 2.7 Hz, 2H), 1.81 (quin, *J* = 7.2 Hz, 2H); ^13^C NMR (101 MHz, DMSO-*d*_6_) δ 159.0 (q, *J* = 31.5 Hz), 140.4, 131.6, 117.6 (q, *J* = 299.1 Hz), 83.3, 72.8, 52.9, 39.1, 21.8, 16.9; ^19^F NMR (376 MHz, DMSO-*d*_6_) δ -73.7; LRMS (ES^+^) *m*/*z* 188 ([M–CF_3_COO]^+^); HRMS (m/z): [M]^+^ calcd. for C_8_H_14_O_2_NS, 188.0740; found, 188.0738.

(*E*)-4-((*tert*-Butoxycarbonyl)amino)but-2-enoic acid **8**^7^

K_2_CO_3_ (196.5 g, 1.424 mol) was suspended in THF (1.0 L). Triethyl phosphonoacetate (319 g, 1.424 mol) was dissolved in THF (200 mL) and added dropwise to the K_2_CO_3_ slurry at rt. **1** (151 g, 0.949 mol) was dissolved in THF (300 mL) and added dropwise to the solution at room temperature. After stirring overnight at rt, water (1.0 L) was added to the solution. The THF was removed by rotary evaporation and the solution was extracted with DCM. The organic layer was washed with brine, dried, concentrated and purified by column chromatography to give (*E*)-ethyl 4-(tert-butoxycarbonylamino)but-2-enoate (120 g, 55%); ^1^HNMR (400 MHz, chloroform-*d*) δ 6.91-6.84 (m, 1H), 5.91-5.86 (m, 1H), 4.16 (q, *J* = 12, 8.0 Hz, 2H), 3.80 (d, *J* = 4.0 Hz, 2H), 3.31-3.29 (m, 1H), 1.44 (s, 9H), 1.28-1.24 (m, 3H).

To a solution of (*E*)-ethyl 4-(tert-butoxycarbonylamino)but-2-enoate (43.46 g, 189.78 mmol) in THF/H_2_O (150 mL/200 mL) was added LiOH^.^H_2_O (11.95 g, 284.67 mmol) dissolved in H_2_O (100 mL) slowly at 0˚C. The reaction mixture was stirred for 5 h, extracted with EtOAc and the water layer was acidified by 2 M HCl to pH 4. The solid was collected by filtration to give **8** (20 g, 52%) as a white solid. ^1^HNMR (300MHz, CDCl3): δ=7.04-6.98 (m, 1H), 5.97-5.92 (m, 1H), 4.75 (s, 1H), 3.95 (s, 2H), 1.46 (s, 9H). The data are in good agreement with literature values.

Pent-4-yn-1-yl (*E*)-4-aminobut-2-enoate hydrochloride **9**

A solution of **8** (8.0 g, 39.80 mmol), 5-chloropent-1-yne (5.309 g, 51.74 mmol), K_2_CO_3_ (7.14 g, 51.74 mmol) and KI (6.605 g, 39.80 mmol) in DMF (150 mL) was stirred at 55^o^C overnight. The solution was diluted with H_2_O and extracted with EtOAc. The organic layer was washed with brine, dried, concentrated and purified by column chromatography to give (*E*)-pent-4-ynyl 4-(tert-butoxycarbonylamino)but-2-enoate (4.0 g, 40%). ^1^HNMR (400MHz, CDCl_3_): δ 6.95-6.88 (m, 1H), 5.95-5.91 (m, 1H), 4.70 (s, 1H), 4.24 (t, *J*=8.0 Hz, 2H), 3.92 (s, 2H), 2.32-2.28 (m, 2H), 1.98-1.97 (m, 1H), 1.96-1.87 (m, 2H), 1.45 (s, 9H); ESI-MS, [M+H]^+^: 268.

A solution of (*E*)-pent-4-ynyl 4-(tert-butoxycarbonylamino)but-2-enoate (6.0 g, 22.45 mmol) was sparged with HCl gas at 0 °C and stirred for 2 h at 0 °C. Concentration gave **9** (4.3 g, 94%) as a yellow solid. ^1^HNMR (400MHz, DMSO-*d*_6_): δ 8.39 (s, 3H), 6.91-6.84 (m, 1H), 6.18-6.13 (m, 1H), 4.19-4.13 (m, 2H), 3.67-3.64 (m, 2H), 2.84 (t, *J*=4.0 Hz, 1H), 2.26-2.23 (m, 2H), 1.81-1.75 (m, 2H); ESI-MS, [M+H]^+^: 168; Purity [HPLC-UV254nm/214nm] > 95%; HRMS (m/z): [M]^+^ calcd. for C_9_H_14_O_2_N, 168.1019; found, 168.1019.

**Probe synthesis**

General methods

All commercial materials were used without further purification. Peptide synthesis reagents (standard amino acid building blocks, PyBop) and Fmoc-l-Leu-l-Ser(ΨMe,Mepro)-OH were purchased from ChemImpex. Fmoc-l-Asp(OtBu)-(Dmb)Gly-OH was purchased from EMD Millipore. Pre-loaded TentaGel Trt R resin (0.18 mmol/g) was purchased from Rapp Polymere GmbH. Solvents were purchased from Biosolve and of HPLC grade or LC-MS grade. LC-MS analysis was performed on a system equipped with a Waters 2795 separation Module (Alliance HT), Waters 2996 Photodiode Array Detector (190−700 nm) and a Micromass LCT-TOF Premier mass spectrometer. Samples were run over an XBridge BEH300 C18 column (5 μm, 4.6×100mm, T= 40°C). Samples were run at 0.8 mL/min using a gradient of two mobile phases: A= 1% acetonitrile and 0.1% formic acid in water; B = 1% water and 0.1% formic acid in acetonitrile. SDS-PAGE analysis was performed on 12% Bolt Bis-Tris gels (LifeTechnologies) and run at 190 V for 30 mins using MES buffer. SDS-PAGE sample preparation: to a 15 μL probe solution (0.2 μg/μL) was added 5 μL 4x Laemmli SDS buffer (containing 7.5% 2-mercaptoethanol) and the sample heated at 90°C for 5 min. Staining is performed with InstantBlue Protein Stain (Expedeon). Preparative HPLC was performed on a Waters XBridge™ Prep C18 column (30×250 mm, 5 μm OBD™). Samples were run at 25 mL/min using a gradient of two mobile phases: A= 5% acetonitrile and 0.05% trifluoracetic acid in water; B= 5% water and 0.05% trifluoroacetic acid in acetonitrile. Data processing was performed using Waters MassLynx 4.1 software.

Ub-VPS


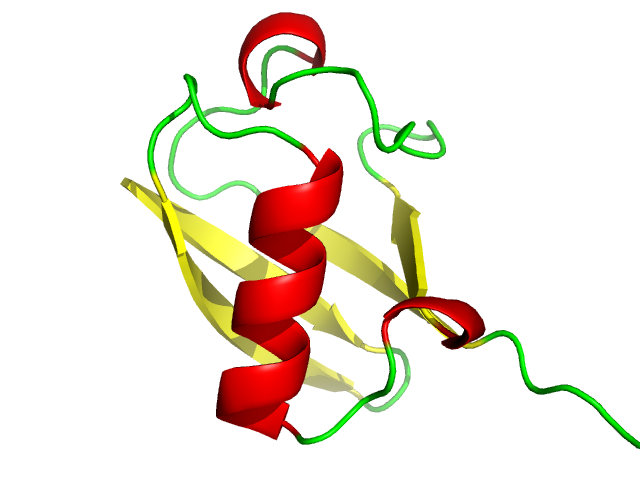


HA-Ahx-Ahx-Ubiquitin(1-75)(Met1Nle)^1^ was synthesized by solid phase peptide synthesis on TentaGel Trt R resin (0.18 mmol/g) as previously reported^8^. The HA-Ahx-Ahx-Ub(1-75) resin (4 µmol) was treated for 1 h with 5 eq Boc_2_O (20 µmol, 4.4 mg) and 10 eq DiPEA (40 µmol, 7 µL). After washing the resin with NMP and DCM, the Boc-HA-Ahx-Ahx-Ub(1-75) was cleaved selectively from the resin by mixing in 15 ml of 4:1 DCM/HFIP for 45 minutes. The resin was flushed 3× with 5 mL DCM and the DCM/HFIP solution was concentrated and co-evaporated 3 × with dichloromethane. The globally protected Boc-HA-Ahx-Ahx-Ub(1-75) was dissolved in 5 mL DCM and treated overnight with PyBOP (40 µmol, 21 mg), vinyl sulfone **7** (25 µmol, 7.5 mg) and DiPEA (80 µmol, 14 µL). The DCM was evaporated and treated for 3 h with 2 mL of TFA/H_2_O/*^i^*Pr_3_SiH/phenol (90/5/2.5/2.5 vol%). The TFA solution was added to 40 mL cold 1:1 *n*-pentane:ether (rinsing reaction flask with 1 mL of TFA) to precipitate the protein. The solution was centrifuged at 2000 rpm for 10 min (with slow brake), the *n*-pentane:ether is decanted and 40 mL ether was added to the protein pellet. The mixture was centrifuged at 2000 rpm for 10 min (with slow brake) and after decanting the ether layer the ether wash step was repeated. The pellet was dissolved in 2.5 mL DMSO, added to 20 mL water and purified by RP-HPLC using the following gradient: 0–6 min: 5–10%B; 6–10 min: 10–30%B; 10–26 min: 30–50%B; 26–27 min: 50–95%B. Lyophilization of pooled fractions afforded 9 mg of HA-Ahx-Ahx-Ub(1-75)-**7** (Ub-VPS) as a white powder (0.9 µmol, 23%).

Ub-VPE


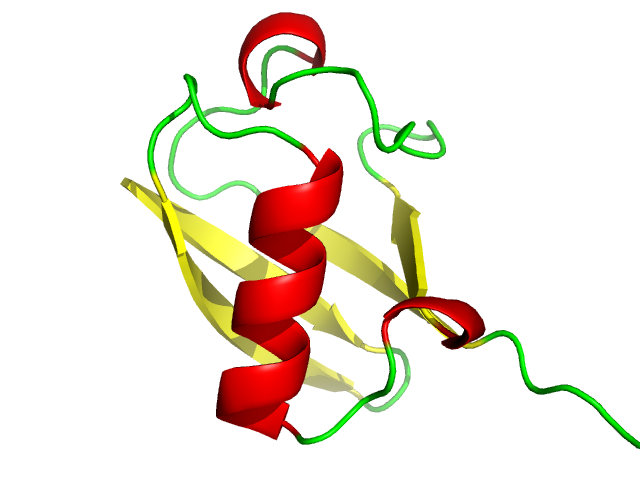


HA-Ahx-Ahx-Ubiquitin(1-75)(Met1Nle)^1^ was synthesized by solid phase peptide synthesis on TentaGel Trt R resin (0.18 mmol/g) as previously reported^8^. The HA-Ahx-Ahx-Ub(1-75) resin (5 µmol) was treated for 1 h with 5 eq Boc_2_O (25 µmol, 5.5 mg) and 10 eq DiPEA (50 µmol, 8.7 µL). After washing the resin with NMP and DCM, the Boc-HA-Ahx-Ahx-Ub(1-75) was cleaved selectively from the resin by mixing in 15 ml of 4:1 DCM/HFIP for 45 minutes. The resin was flushed 3 × with 5 mL DCM and the DCM/HFIP solution was concentrated and co-evaporated 3 × with dichloromethane. The globally protected Boc-HA-Ahx-Ahx-Ub(1-75) was dissolved in 5 mL DCM and treated overnight with PyBOP (25 µmol, 13 mg), vinyl ester **9** (20 µmol, 4 mg) and DiPEA (50 µmol, 8.7 µL). The DCM was evaporated and treated for 3 h with 2 mL of TFA/H_2_O/iPr_3_SiH/phenol (90/5/2.5/2.5 vol%). The TFA solution was added to 40 ml cold 1:1 *n*-pentane:ether (rinsing reaction flask with 1 mL of TFA) to precipitate the protein. The solution was centrifuged at 2000 rpm for 10 min (with slow brake), the *n*-pentane:ether was decanted and 40  mL ether was added to the protein pellet. The mixture was centrifuged at 2000 rpm for 10 min (with slow brake) and after decanting the ether layer the ether wash step was repeated. The pellet was dissolved in 2.5 mL DMSO, added to 20 mL water and purified by RP-HPLC using the following gradient: 0–6 min: 5–10%B; 6–10 min: 10–30%B; 10–26 min: 30–50%B; 26–27 min: 50–95%B. Lyophilization of pooled fractions afforded 10 mg of HA-Ahx-Ahx-Ub(1-75)-**9** (Ub-VPE) as a white powder (1 µmol, 25%).

SUMO2-VPS


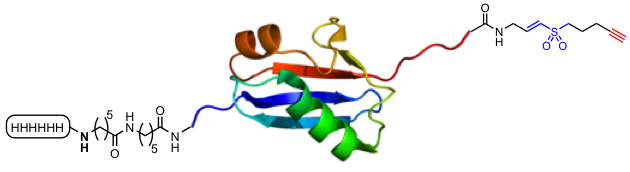


His_6_-Ahx-Ahx-SUMO2(1-92)(C48S) was synthesized on a Syro II MultiSyntech Automated Peptide synthesizer using standard 9-fluorenylmethoxycarbonyl (Fmoc) based solid phase peptide chemistry at 2 µmol scale using the Tip synthesis module and a fourfold excess of amino acids relative to Fmoc-Gly loaded TentaGel Trt R resin (0.18 mmol/g). All cycles were performed with double couplings in NMP for 25 min using PyBOP (4 equiv) and DiPEA (8 equiv). For the final 6 *N*-terminal His residues we used double couplings in NMP for 45 min. The His_6_ tag is separated from the *N*-terminus by two aminohexanoic acid (Ahx) linkers for efficient recognition of the His_6_ tag. Fmoc removal was performed with 20% piperidine in NMP for 3 and 5 min. The following protected amino acids were used Fmoc-L-Ala-OH, Fmoc-L-Arg-(Pbf)-OH, Fmoc-L-Asn(Trt)-OH, Fmoc-L-Asp(OtBu)-OH, Fmoc-L-Gln(Trt)-OH, Fmoc-L-Glu(OtBu)-OH, Fmoc-L-Gly-OH, Fmoc-L-His(Trt)-OH, Fmoc-L-Ile-OH, Fmoc-L-Leu-OH, Fmoc-L-Lys(Boc)-OH, Fmoc-L-Met-OH; Fmoc-L-Phe-OH; Fmoc-L-Pro-OH; Fmoc-L-Ser(tBu)-OH; Fmoc-L-Thr(tBu)-OH, Fmoc-L-Tyr(tBu)-OH and FmocL-Val-OH. Pseudoproline Fmoc-L-Leu-L-Ser(ΨMe,Mepro)-OH was incorporated at position Leu53-Ser54. The 2,4-dimethoxybenzyl protected dipeptide Fmoc-L-Asp(OtBu)-(Dmb)Gly-OH was incorporated at positions Asp26-Gly27 and Asp63-Gly64. All amino acid and dipeptide building blocks were dried overnight under high vacuum prior to use. After the synthesis the resin was washed with diethylether and dried under high vacuum. Next, the polypeptide sequence was detached from an aliquot of the resin (2-3 mg) and deprotected by treatment with 100 µL TFA/H_2_O/*^i^*Pr_3_SiH/phenol (90/5/2.5/2.5 vol%) for 3 h. The TFA solution was added to 1 mL cold 1:1 *n*-pentane:ether to precipitate the protein. The solution was centrifuged at 13000 rpm for 10 min, the *n*-pentane:ether was decanted and 1 mL ether was added to the protein pellet. The crude His_6_-Ahx-Ahx-SUMO2(1-92)(C48S) was analyzed by LC-MS (Supplementary Figure 3e).

The remaining His_6_-Ahx-Ahx-SUMO2(1-92)(C48S) resin (4 µmol) was treated for 1 h with 5 eq Boc_2_O (20 µmol, 4.4 mg) and 10 eq DiPEA (40 µmol, 7 µL). After washing the resin with NMP and DCM, the Boc-His_6_-Ahx-Ahx-SUMO2(1-92)(C48S) was cleaved selectively from the resin by mixing in 15 ml of 4:1 DCM/HFIP for 45 minutes. The resin was flushed 3× with 5 mL DCM and the DCM/HFIP solution was concentrated and co-evaporated 3× with dichloromethane. The globally protected Boc-His_6_-Ahx-Ahx-SUMO2(1-92)(C48S) was dissolved in 5 mL DCM and treated overnight with PyBOP (40 µmol, 21 mg), vinyl sulfone **7** (25 µmol, 7.5 mg) and DiPEA (80 µmol, 14 µL). The DCM was evaporated and treated for 3 h with 1.5 mL of TFA/H_2_O/*^i^*Pr_3_SiH/phenol (90/5/2.5/2.5 vol%). The TFA solution was added to 40 mL cold 1:1 *n*-pentane:ether (rinsing reaction flask with 1 mL of TFA) to precipitate the protein. The solution was centrifuged at 2000 rpm for 10 min (with slow brake), the *n*-pentane:ether was decanted and 40 mL ether was added to the protein pellet. The mixture was centrifuged at 2000 rpm for 10 min (with slow brake) and after decanting the ether layer the ether wash step was repeated. The pellet was dissolved in 2.5 mL DMSO, added to 20 mL water and purified by RP-HPLC using the following gradient: 0–6 min: 5–10%B; 6–10 min: 10–30%B; 10–26 min: 30–50%B; 26–27 min: 50–95%B. Lyophilization of pooled fractions afforded 23 mg of His_6_-Ahx-Ahx-SUMO2(1-92)(C48S)-VPS (SUMO2-VPS) as a white powder (23 mg, 1.9 µmol, 49%).

**Supplementary References**

1. Xu, L. *et al.* Efficient chemical synthesis for the analogue of ubiquitin-based probe Ub–AMC with native bioactivity. *RSC Adv.* **6,** 47926–47930 (2016).

2. Robert, X. & Gouet, P. Deciphering key features in protein structures with the new ENDscript server. *Nucleic Acids Res* **42,** W320–4 (2014).

3. Kathman, S. G., Xu, Z. & Statsyuk, A. V. A Fragment-Based Method to Discover Irreversible Covalent Inhibitors of Cysteine Proteases. *J Med Chem* **57,** 4969–4974 (2014).

4. Benati, L. *et al.* Generation and Intramolecular Reactivity of Acyl Radicals from Alkynylthiol Esters under Reducing Tin-Free Conditions. *Org Lett* **5,** 1313–1316 (2003).

5. Wang, G. & Yao, S. Q. Combinatorial synthesis of a small-molecule library based on the vinyl sulfone scaffold. *Org Lett* **5,** 4437–4440 (2003).

6. Claridge, T. D. W. *et al.* Highly ( E)-Selective Wadsworth−Emmons Reactions Promoted by Methylmagnesium Bromide. *Org Lett* **10,** 5437–5440 (2008).

7. McGouran, J. F. *et al.* Fluorescence-based active site probes for profiling deubiquitinating enzymes. *Org Biomol Chem* **10,** 3379–3383 (2012).

8. Oualid, El, F. *et al.* Chemical Synthesis of Ubiquitin, Ubiquitin‐Based Probes, and Diubiquitin. *Angew Chem Int Ed Engl* **49,** 10149–10153 (2010).
