# Supplementary material for: Reactive-site-centric chemoproteomics identifies a distinct class of deubiquitinase enzymes
Source: Nat Commun. 2018 Mar 21;9:1162. doi: 10.1038/s41467-018-03511-6 (PMC5862848; doi:10.1038/s41467-018-03511-6)
Supplement: Supplementary file 3 — Description of Additional Supplementary Files(PDF 82 kb) [file 41467_2018_3511_MOESM3_ESM.pdf]

Descriptions of Additional Supplementary Files:

File Name: Supplementary Data 1
Description: Peptide-spectrum matches under different enrichment/digestion conditions. Shown is the total number of peptide-spectrum matches (PSMs), with the number of unique peptides in brackets. Red shading indicates that ≥3 unique peptides were identified (criterion for counting in Figure 3a).

File Name: Supplementary Data 2
Description: Proteins detected in on-bead digestion fractions. Sheets 1 and 2 show the total number of peptide-spectrum matches (PSMs), with the number of unique peptides in brackets. Red shading indicates that ≥3 unique peptides were identified.

File Name: Supplementary Data 3
Description: Comparison of DUB detection by ABPP with RNA expression. Shown are RPKM values (from RNA-Seq) and feature count (derived from ABPP experiment with Ub-VPS, three biological replicates). Proteins with pink shading are expressed (RPKM > 1) but not detected by ABPP.

File Name: Supplementary Data 4
Description: Ub-VPS labeling sites identified by reactive-site-centric chemoproteomics. Shown is the total number of peptide-spectrum matches (PSMs) for each labeling site across the indicated digestion conditions and six replicates (indicated by Roman numerals). Gray shading: 0 PSMs; yellow shading: 1 PSM, red shading: ≥2 PSMs. *: non-catalytic Cys residue of DUB; †: catalytic residue of non-DUB.

File Name: Supplementary Data 5
Description: NMR chemical shift changes in K48- and K63-linked di-Ub upon addition of ZUFSP. Residues which undergo substantial chemical shift change or line broadening upon ZUFSP addition are indicated by shading: red for distal ubiquitin (15N labeled), green for proximal ubiquitin (13C labeled).

File Name: Supplementary Data 6
Description: Affinity purification-mass spectrometry analysis of FLAG-ZUFSP. Shown is the number of peptide-spectrum matches (PSMs), with the number of unique peptides in brackets. RPA proteins are highlighted in red.
